# Spread and establishment of *Aedes albopictus* in southern Switzerland between 2003 and 2014: an analysis of oviposition data and weather conditions

**DOI:** 10.1186/s13071-016-1577-3

**Published:** 2016-05-26

**Authors:** Eleonora Flacio, Lukas Engeler, Mauro Tonolla, Pie Müller

**Affiliations:** Laboratory of Applied Microbiology, University of Applied Sciences and Arts of Southern Switzerland, via Mirasole 22A, 6500 Bellinzona, Switzerland; Laboratory of Eco-Epidemiology of Parasites, Institute of Biology, University of Neuchâtel, Emile-Argand 11, 2000 Neuchâtel, Switzerland; Microbiology Unit, Plant Biology Department, Sciences III University of Geneva, Quai Ernest-Ansermet 30, 1211 Geneva, Switzerland; Department of Epidemiology and Public Health, Swiss Tropical and Public Health Institute, Socinstrasse 57, PO Box, 4002, Basel, Switzerland; University of Basel, Petersplatz 1, 4003 Basel, Switzerland

## Abstract

**Background:**

The Asian tiger mosquito, *Aedes albopictus*, is a highly invasive mosquito species of public health importance. In the wake of its arrival in neighbouring Italy the authorities of the canton of Ticino in southern Switzerland initiated a surveillance programme in 2000 that is still on-going. Here we explored the unique data set, compiled from 2003 to 2014, to analyse the local dynamic of introduction and establishment of *Ae. albopictus*, its relative density in relation to precipitation and temperature, and its potential distribution at the passage from southern to northern Europe.

**Methods:**

The presence of *Ae. albopictus* was recorded by ovitraps placed across Ticino. In addition to presence-absence, the relationship between relative egg densities and year, month, temperature and precipitation was analysed by a generalised linear mixed model.

**Results:**

Since its first detection in 2003 at Ticino’s border with Italy *Ae. albopictus* has continuously spread north across the lower valleys, mainly along the trans-European motorway, E35. Detailed local analysis showed that industrial areas were colonised by the mosquito before residential areas and that, afterwards, the mosquito was more present in residential than in industrial areas. *Ae. albopictus* appeared sporadically and then became more present in the same places the following years, suggesting gradual establishment of locally reproducing populations that manage to overwinter.

This trend continues as witnessed by both a growing area being infested and increasing egg counts in the ovitraps. There was a clear South-North gradient with more traps being repeatedly positive in the South and fewer eggs laid during periods of intensive precipitation. In the North, the mosquito appeared repeatedly through the years, but never managed to establish, probably because of unfavourable weather conditions and low road traffic.

**Conclusions:**

Given the present results we assume that additional areas may still become infested. While the current study provides good estimates of relative egg densities and shows the local and regional dynamics of *Ae. albopictus* invasion, additional parameters ought to be measured to make an objective risk assessment for epidemic disease transmission. The likelihood of *Ae. albopictus* to further spread and increase in densities calls for continued surveillance.

**Electronic supplementary material:**

The online version of this article (doi:10.1186/s13071-016-1577-3) contains supplementary material, which is available to authorized users.

## Background

The Asian tiger mosquito, *Aedes albopictus* (Skuse, 1894), is considered the most invasive mosquito species worldwide [1, 2]. During the past 40 years *Ae. albopictus* spread from South-East Asia to North and South America, parts of Africa, northern Australia, several Pacific and Oceanic islands as well as many European countries [3, 4]. Besides being an invasive species from an environmental point of view, this mosquito also threatens human and animal health. *Ae. albopictus* is a vector of chikungunya, dengue, zika virus as well as dirofilarial worms and, under laboratory conditions, is able to transmit at least 27 arboviruses [3, 5]. In continental Europe, several autochthonous cases of chikungunya in Italy and France [6–8], and dengue in Croatia and France [9–13], have been associated with *Ae. albopictus*.

It is assumed that the active flight range of *Ae. albopictus* is only a few hundred metres. For example, Marini et al. [14] reported that the mosquito’s average daily flight distance is only 119 m, while, owing to its desiccation resistant eggs, *Ae. albopictus* is passively spread over long distances through the international trade of used tyres and other artificial containers. At a more regional level, adult mosquitoes are frequently stowed away in vehicles and subsequently displaced along roads [3, 15].

In Europe, *Ae. albopictus* was first recorded in Albania in 1979 [16] and later in Italy, in 1990 [17]. Less than a decade later it was established in the northern and central regions of Italy [18, 19] from where it spread further across Europe by means of public and private transport [20]. *Ae. albopictus* is currently established in most Mediterranean coastal regions, including the islands, from Alicante in Spain to Athens in Greece and across the whole of Italy [4, 21]. In the North, the mosquito has already been reported close to Paris and Strasbourg [22] and southern Germany along two motorways connecting with southern Europe and in the cities of Heidelberg and Freiburg [23–25].

Fearing the possibility of *Ae. albopictus* introduction from northern Italy and the associated risk of biting nuisance and disease transmission, the authorities of the Republic and Canton of Ticino, a southern Swiss region bordering with Italy, initiated the surveillance of *Ae. albopictus* in the year 2000. In the beginning, surveillance focused on the trans-European motorway E35 that runs through Ticino, connecting Italy with northern Europe. *Ae. albopictus* was first observed at a motorway service area in 2003 [26]. Since then the surveillance programme, consisting of *Ae. albopictus* monitoring and control, has been continuously expanded. The details of set-up and the history of the Ticino surveillance programme are described in Flacio et al. [27].

Based on oviposition data collected between 2003 and 2014 we describe the local dynamic of introduction, presence and establishment of *Ae. albopictus* and its relative density in relation to precipitation and temperature in southern Switzerland.

## Methods

### Study area

**Fig. 1:**
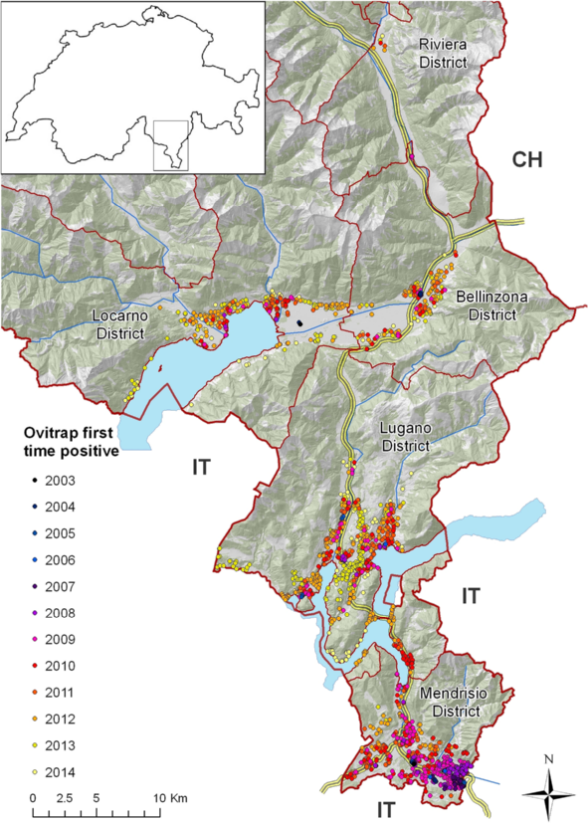
Spatial and temporal distribution of *Aedes albopictus* in the Canton of Ticino since its introduction to Switzerland. Dot colours indicate when the ovitraps were first positive to *Ae. albopictus*. The yellow lines show the motorways. CH: Switzerland; IT: Italy. Map layers were purchased from the Swiss Federal Office of Topography

Data were collected in the Canton of Ticino, Switzerland, between 3 July 2003 and 22 September 2014. The Canton of Ticino is located south of the Alps and is divided into eight districts five of which were considered in this study: Mendrisio, Lugano, Locarno, Bellinzona and Riviera (Fig. 1). The landscape is dominated by agriculture and forested hills, interspersed with lakes, rivers and mountains that culminate at more than 3.000 m above sea level (a.s.l). Urban settlements are mainly characterised by two-story houses, surrounded with private gardens and are mostly located in valley floors below 400 m a.s.l. [28].

Over 62,000 commuters (data from 2014 [29]) cross the border between Italy and Ticino on a work day. The canton is also an important passage for long distance road traffic with the trans-European motorway E35 that runs from Rome (Italy) to Amsterdam (the Netherlands) through Ticino (Fig. 1).

### Mosquito trapping

All data were collected in the frame of a routine surveillance programme that was initiated in the year 2000 and then gradually expanded to cover larger areas in response to reported and suspected presence of *Ae. albopictus*. The details of the programme and how it evolved over the years are described in Flacio et al. [27].

Briefly, *Ae. albopictus* surveillance started by placing a few oviposition traps, hereafter called “ovitraps”, at suspected entry points near the border with Italy such as motorway service areas and locations with potential breeding sites like cemeteries with flower vases. Between 2005 and 2008, the surveillance was gradually expanded, including industrial zones and larger car parks and public areas in cities. In 2009 the initially targeted monitoring was extended to an area-wide surveillance network covering the urban areas of entire municipalities [27].

Ovitraps consisted of 1.5 l black plastic containers filled with tap water into which a wooden slat is placed as an oviposition substrate. To avoid larval development inside the trap, *Bacillus thuringiensis* var. *israelensis* (*Bti*) granules were added to the water (for details see [27]). Ovitraps were positioned on the ground under vegetation or near buildings, and slats, water and *Bti* granules were replaced bi-weekly.

### Data recording

The wooden slats were brought to the laboratory in Canobbio near Lugano and carefully inspected under a stereo microscope with an 80x magnification. Eggs were morphologically identified according to Zamburlini & Frilli [30] and counted. Morphological identification was regularly controlled by hatching out eggs and by rearing the mosquitoes to late larval instars or adults to confirm the species. Since 2013 batches of eggs have also been identified using Matrix Assisted Laser Desorption/Ionization - Time of Flight Mass Spectrometry (MALDI-TOF MS) approach [31].

Egg counts together with associated information, including trap position, date of collection and trap condition were entered into an Access 2000 database (Microsoft Corporation Ltd., USA).

### Distribution and annual activity patterns

Survey periods varied between years, ranging between calendar week 14 and 48 but always covered the *Ae. albopictus* peak season (Additional file 1). *Aedes albopictus* females lay eggs that undergo diapause during winter in more temperate regions [32]. To determine when overwintering eggs hatch, slats with *Ae. albopictus* eggs that have been collected from positive ovitraps in calendar week 40 in 2009 were left in the field. The slats were kept in mosquito breeders (similar to the ones available on www.bioquip.com) made of 1,5 l transparent PET bottles. These breeders were then placed inside the black ovitrap container. Mosquito breeders were checked for the presence of larvae bi-weekly until hatching in 2010 [33].

In addition, to monitor mosquito activity during winter, ovitraps in locations that previously showed high egg counts were left over winter and inspected each month for the presence of eggs. Eight ovitraps were set in Lugano and eight traps in Chiasso from December to April 2012–2013 and 2013–2014.

### Analysis of egg counts

For comparison between years, only data from the calendar weeks 22, 26, 30, 34 and 38 (May - September) were considered (Additional file 1) to account for variations in the length of the sampling periods between years. Depending on whether a trap was negative, sporadically or continuously positive during three consecutive 4-week periods, *Ae. albopictus* was deemed “absent”, “introduced” or “established”, respectively. If an ovitrap was positive both in calendar week 38 and in calendar week 22 of the following year, then the local population was assumed to have overwintered in that location. To visualise these, data maps were drawn using ArcMap 10.2.2 (ESRI Inc., USA).

In addition to the presence-absence of *Ae. albopictus,* we also investigated the relationship between actual egg counts and several potential covariates by fitting a generalised linear mixed model (GLMM) with a negative binomial link function. In the GLMM the dependent variable was the total number of eggs on a single slat, while the explanatory variables considered were the year and month of collection, temperature and precipitation. These covariates were included as fixed effects, while a random intercept was added for the ovitraps, accounting for repeated measures in the same ovitrap. In the analysis, the weekly average temperature preceding 1, 2, 3 or 4 weeks as well as the average temperature over 1, 2, 3 or 4 weeks prior inspecting the traps were considered. For precipitation (i.e. rain) the cumulative volume per area was computed. Similar to temperature, the weekly sum preceding 1, 2, 3 or 4 weeks as well as the cumulative precipitation over 1, 2, 3 or 4 weeks prior inspecting the traps were considered. From the full model including all above covariates only those that were significant (α = 0.5) were retained in the final model. Moreover, only one term for each temperature and precipitation was included in the model. Meteorological data were retrieved from eight stations present in the study area (Additional file 2).

Data analysis of egg counts was done using the open source software package R version 3.0.2 [34] with the “glmmadmb()” function from the glmmADMB package [35, 36].

## Results

### *Aedes albopictus* activity period

**Fig. 2:**
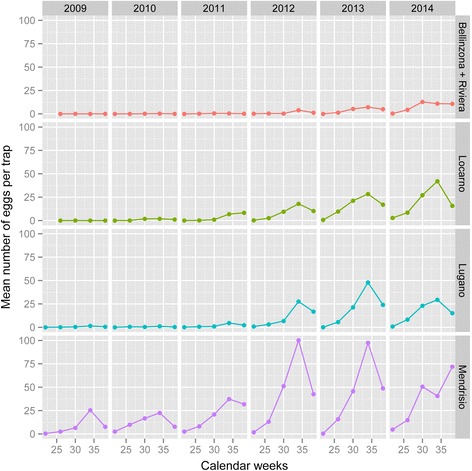
Mean *Aedes albopictus* egg numbers per trap according to districts between 2009 and 2014. The graphs show the average egg numbers per trap by year and calendar week

Newly laid *Ae. albopictus* eggs were found only during or after calendar week 22 in mid-May (i.e. the ovitraps were set in week 20), while eggs were still collected up to the calendar week 48 in mid-November when day length was 10 h and the mean temperature was 7.6 °C. During mild falls, when the mean temperature was still around 9 °C, females continued to lay non-diapausing eggs and fourth instar larvae were found in mid-November. Maximum egg numbers were generally recorded in August in calendar week 34 (Fig. 2) when the mean temperature was 21.1 °C. Eggs left outdoors remained in diapause over the winter until mid- to end-April (i.e. calendar week 16 or 18) when day length was 11 to 11.5 h and the mean temperature was 12.3 °C. In summary, *Ae. albopictus* reached its activity peak in August, while the eggs went into diapause from mid-November to mid-April.

### Temporal and local distribution of *Aedes albopictus*

A general overview on *Ae. albopictus* occurrence during the year is shown in Fig. 1, whereas detailed maps in Additional file 3 describe the occurrence, establishment and overwintering of the mosquito year by year. In 2003, four out of 166 slats (4.2 %) were positive in Mendrisio and Locarno districts (Fig. 1, Additional files 3 and 4). Over the following years a steady increase in the number of positive ovitraps was observed till 2007, when *Ae. albopictus* seemed to have established the first seasonally stable population in the border town Chiasso and later also in the neighbouring villages (Mendrisio district). While Mendrisio was the first widely infested district, the presence of the mosquito in the remaining districts was still patchy and of transient nature at the time (Fig. 1, Additional file 3). The next turning point took place in 2008 (Fig. 1, Additional files 3 and 4) when *Ae. albopictus* was repeatedly observed in residential areas in the Lugano and Locarno districts. Between 2009 and 2011, the mosquito continuously extended its range (Fig. 1 and Additional file 3) and in 2012 its presence significantly increased in many locations across the canton (Fig. 1, Additional files 3 and 4). In 2009, the first overwintering populations were observed at the border region in Mendrisio district and in 2011 in the Lugano district (Additional file 3). During the last two study years, changes were less dramatic, although the trend of *Ae. albopictus* expansion continued in Ticino.

**Fig. 3:**
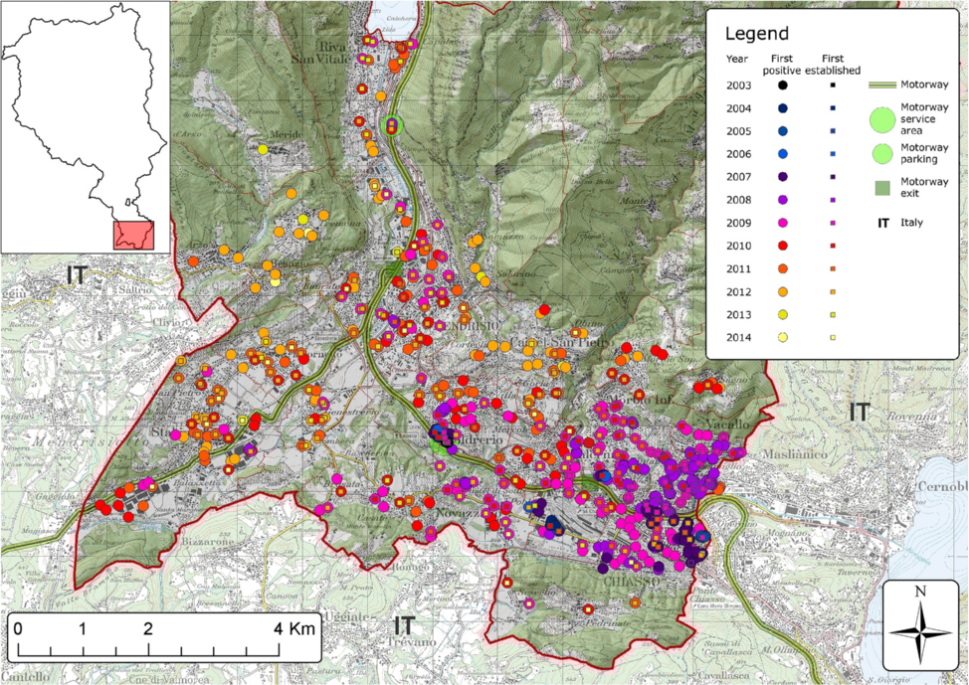
Detailed view of *Ae. albopictus* first detections and first evidences of establishment on ovitraps in the Mendrisio district from 2003 to 2014. Dots represent an ovitrap and the colour indicates the year when ovitraps were positive for *Ae. albopictus* the first time. The squares indicate the year in which *Ae. albopictus* was considered to have established a local population. For this an ovitrap had to be positive for three consecutive 4 week periods. Map layers were purchased from the Swiss Federal Office of Topography

**Fig. 4:**
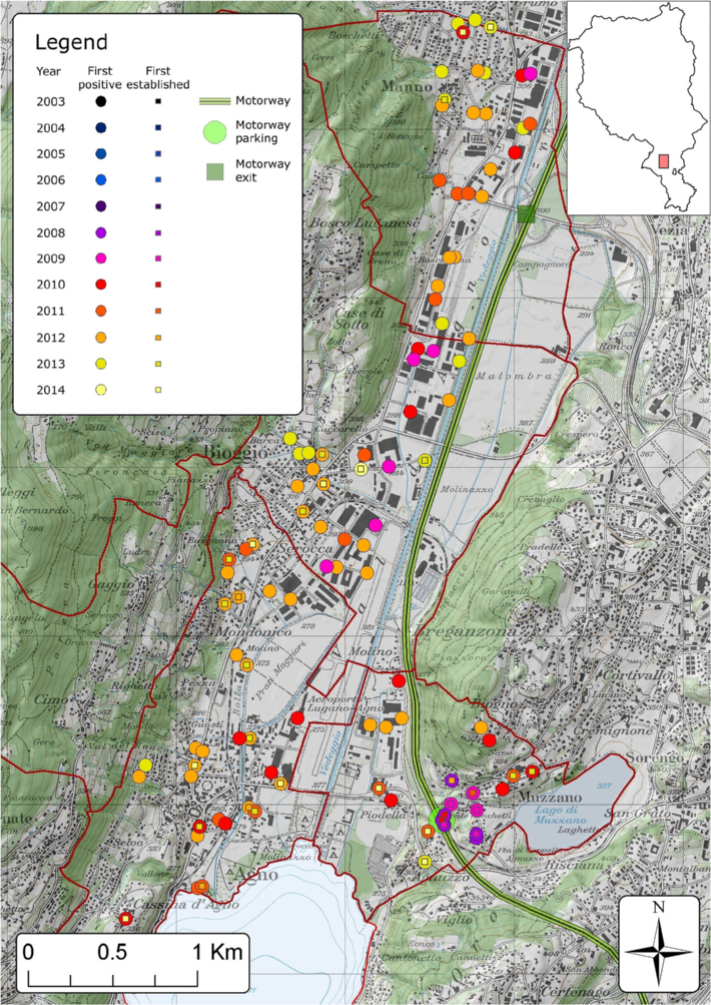
Detailed view of *Ae. albopictus* first detections and first evidences of establishment on ovitraps in the Lugano district from 2003 to 2014. Dots represent an ovitrap and the colour indicates the year when ovitraps were positive for *Ae. albopictus* the first time. The squares indicate the year in which *Ae. albopictus* was considered to have established a local population. For this an ovitrap had to be positive for three consecutive 4 week periods. Map layers were purchased from the Swiss Federal Office of Topography

**Fig. 5:**
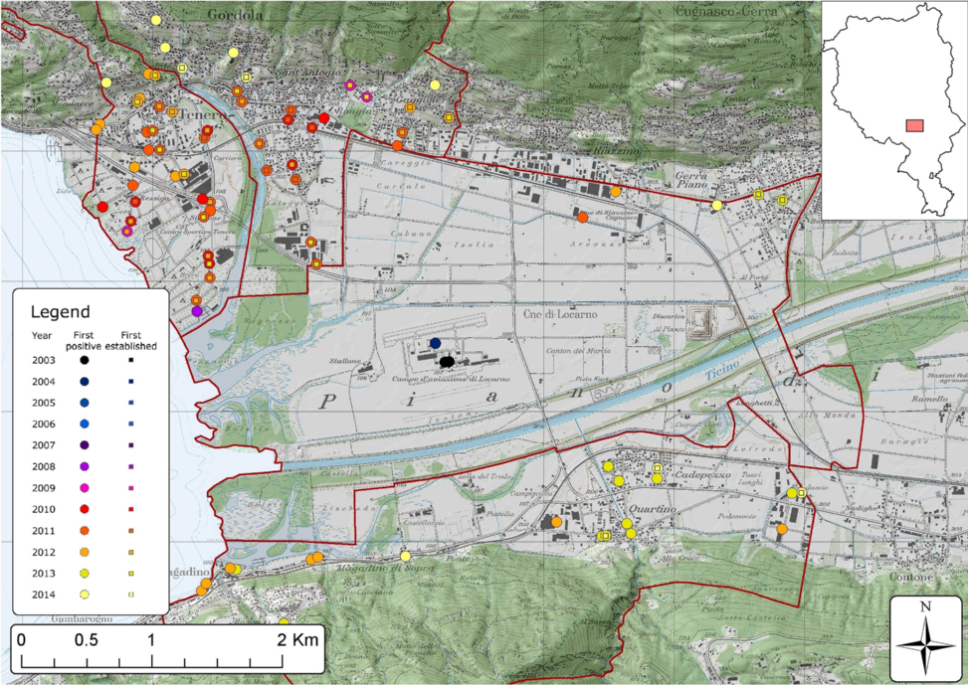
Detailed view of *Ae. albopictus* first detections and first evidences of establishment on ovitraps in the Locarno district from 2003 to 2014. Dots represent an ovitrap and the colour indicates the year when ovitraps were positive for *Ae. albopictus* the first time. The squares indicate the year in which *Ae. albopictus* was considered to have established a local population. For this an ovitrap had to be positive for three consecutive 4 week periods. Map layers were purchased from the Swiss Federal Office of Topography

**Fig. 6:**
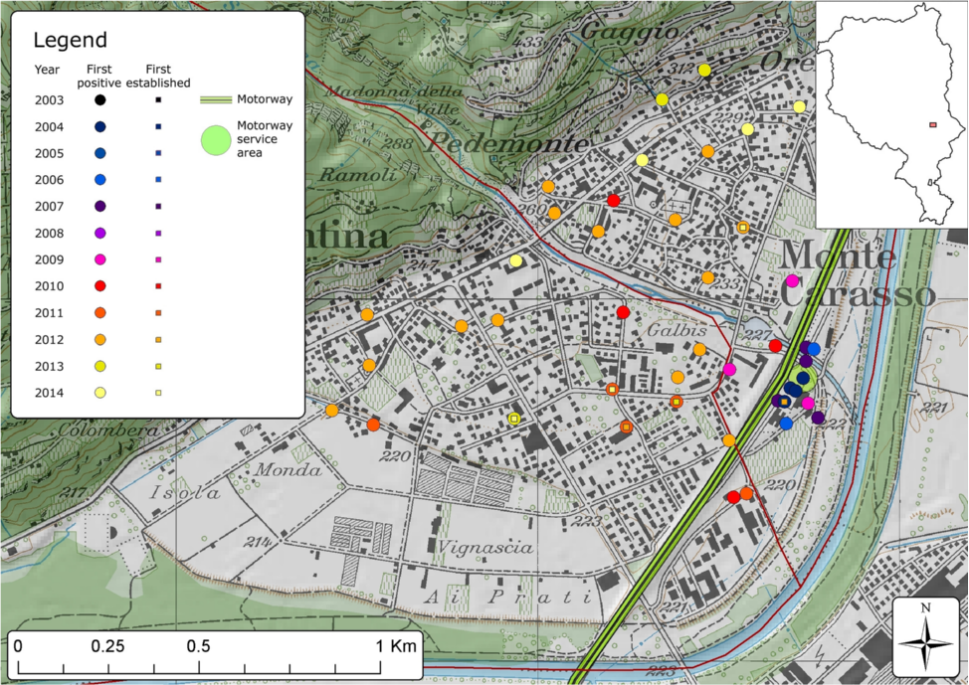
Detailed view of *Ae. albopictus* first detections and first evidences of establishment on ovitraps in the Bellinzona district from 2003 to 2014. Dots represent an ovitrap and the colour indicates the year when ovitraps were positive for *Ae. albopictus* the first time. The squares indicate the year in which *Ae. albopictus* was considered to have established a local population. For this an ovitrap had to be positive for three consecutive 4 week periods. Map layers were purchased from the Swiss Federal Office of Topography

The detailed local dynamic of how *Ae. albopictus* spread and established in various urban areas across Ticino is shown in Figs. 3, 4, 5 and 6. Service stations and parking areas along the motorway appeared to be one of the first introduction points in the territory (Figs. 3, 4 and 6). They were constantly infested by *Ae. albopictus*. In these areas the mosquito clearly managed to establish before its establishment in other urban settlements.

In 2003, two of the first positive slats appeared at the first E35 motorway service area after the border from Italy in the Mendrisio district (Coldrerio East) (Figs. 1 and 3). The border control area in Chiasso in the south-eastern part of Mendrisio district was also one of the first points of *Ae. albopictus* detection. Furthermore traps positioned in the area close to the motorway exit of Mendrisio were positive before other traps in the same area. In this district, the established mosquito population continued to increase over time (Figs. 1 and 3).

In the Lugano district the first signs of establishment of *Ae. albopictus* appeared in residential areas close to a motorway parking place in 2009 (Fig. 4). In the rest of the district the occurrence of the mosquito increased constantly and in 2010 and 2011 some quarters of Lugano, the largest city of the Canton, were infested (Fig. 1 and Additional file 3). Similarly, in the Locarno district there was a progressive mosquito occurrence, with the first signs of establishment and overwintering in 2011 (Fig. 5). Interestingly, two of the first positive slats in 2003 were detected at the Locarno Airport (Locarno district), and again during summer 2004, but could not be detected during the following survey seasons thanks to repeated application of *Bti* and permethrin. The mosquito was then detected again in 2008 and 2009 in camping sites closed to this area.

In the Bellinzona district, the mosquito appeared in residential areas in 2009 and then constantly increased, even if its presence remained lower than in the other districts. *Ae. albopictus* established in the North of the district only in 2012 (Fig. 1 and Additional file 3). Further north, in the municipality of Biasca (Riviera district), the tiger mosquito appeared repeatedly through the years, but never managed to establish (Fig. 1 and Additional file 3).

Interestingly, industrial areas were apparently colonised by the mosquito before residential areas with a lead time of 2 to 3 years as observed in Agno, Bioggio and Manno municipalities in the Lugano district (Fig. 4) as well as in Stabio municipality in the Mendrisio district at the southwest border with Italy (Fig. 3). However, from then on the mosquito was more present in residential than in industrial areas (Figs. 3 and 4).

When ovitraps were place at altitudes above 400 m, establishment was delayed (Fig. 3).

In summary, since 2003 *Ae. albopictus* has spread across Ticino from South to North mainly along the trans-European motorway E35. We observed a massive surge around 2011 and 2012 and *Ae. albopictus* still continues its spread.

### Weather conditions during the annual surveys

Consistent data from the eight weather stations were available between 2006 and 2014. Seasonal and annual mean temperatures recorded at each weather station are summarized in Additional file 2. The annual mean temperature in the study area was 12.5 °C with a mean summer temperature of 19.5 °C during the surveys between May and September. July was the warmest month, with a mean temperature of 22 °C with minimum temperatures ranging from 8.5 °C to 11.1 °C and maximum temperatures ranging from 33.7 °C to 38.5 °C. January was the coldest month with a mean temperature of 2.9 °C, with minimum temperatures ranging from -10.7 °C to 14.5 °C and maximum from -3.0 °C to 24.8 °C. Mean among the weather stations of total annual rainfall was 1789 mm, whereas mean of total precipitation during the survey season was 857 mm. The wettest mosquito season (May-September) occurred in 2008 with a total rainfall of 8856 mm and the driest occurred in 2013 with 5816 mm of cumulated rain. The total number of days with minimum temperature below -10 °C, that is the minimum temperature considered for the survival of diapausing eggs [37, 38], between 2006–2014 varied from 0 to 14 (Additional file 2).

### Relative egg densities

**Fig. 7:**
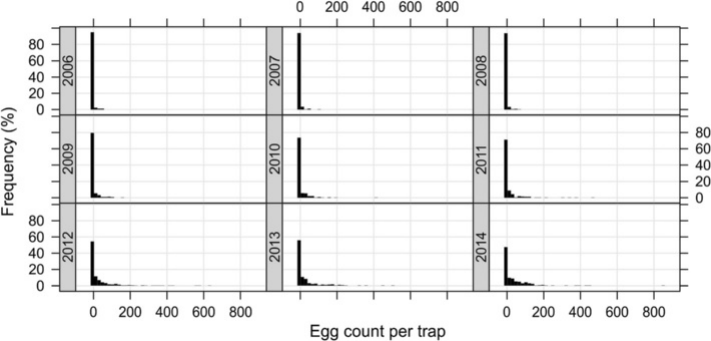
Egg numbers in sentinel sites between 2006 and 2014. The histograms show the percentage frequencies of egg counts in the 46 sentinel traps present throughout 2006 to 2014

**Fig. 8:**
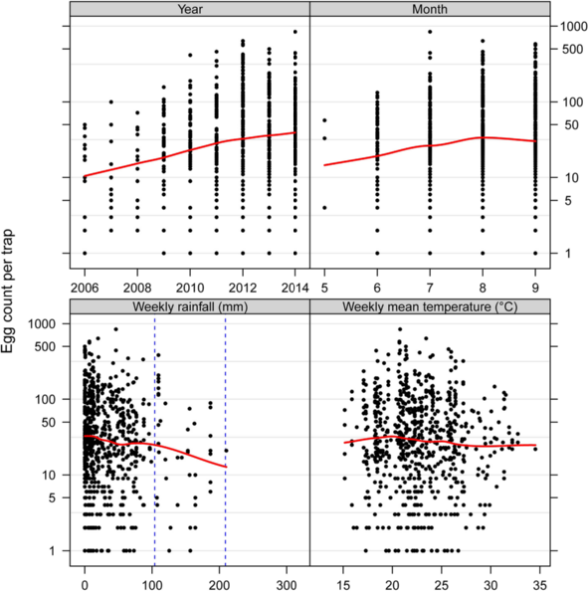
Relationship between egg numbers and covariates in the sentinel traps of the Ticino *Aedes albopictus* surveillance since 2006. The multi-panel scatterplots show the egg counts on each slat from the 46 sentinel ovitraps as a function of the four covariates; year, month, rain and temperature. Rain was calculated as the cumulative precipitation over the week before collecting the slat and temperature accordingly as the mean temperature during the preceding week. A LOESS smoother (red line) was added to aid visual interpretation. The visual inspection indicates an increase in egg counts over the years and a seasonal maximum in August. The variable “rain” was split into three levels, represented by the vertical blue stippled lines. The levels were “low”, “middle” and “high” with 0–104 mm, over 104 to 209 mm and over 209 to 314 mm, respectively. Note that egg counts are plotted on a logarithmic scale; and hence zero counts are not shown due to points at infinity

As there were only very few trap locations consistently present since 2003 only data from ovitraps set between 2006 and 2014 were included in the relative egg density analysis. Forty-six traps were always present between 2006 and 2014 (Additional file 5). In these 46 traps a total of 3,358 egg collections were made. Due to lost or damaged traps 370 records had to be removed from the analysis. From the analysed slats we found that the frequency of zero counts decreased as of 2009, while most of the trap counts were still zeros in 2006 (Fig. 7). From 2009, increasingly larger counts were recorded across the 46 traps. One trap had 844 eggs in 2014. This trend is also reflected in the GLMM model in that the factor “year” shows increasing ratios for the estimates over the years as compared to the year 2006 (Fig. 8 and Table 1). Compared to 2006 the relative density in 2014 has increased 87.4 times from an average biweekly egg count of 0.003 eggs per trap to 0.262 eggs per trap.

**Table 1:** Result summary for the negative binomial model for the relationship between egg counts, year, month and precipitation for the years 2006 to 2014

Predictor	Coefficient β (log_2_)	SE(β) (log_2_)	*Z*-value	*P*-value
Intercept	-5.81	0.596	-9.75	< 0.001
2007	1.278	0.374	3.42	< 0.001
2008	0.292	0.36	0.81	ns
2009	1.88	0.341	5.51	< 0.001
2010	2.609	0.369	7.08	< 0.001
2011	2.801	0.345	8.13	< 0.001
2012	4.383	0.355	12.36	< 0.001
2013	3.935	0.366	10.75	< 0.001
2014	4.471	0.358	12.47	< 0.001
June	2.745	0.467	5.88	< 0.001
July	4.402	0.468	9.4	< 0.001
August	5.125	0.464	11.03	< 0.001
September	5.1	0.471	10.83	< 0.001
Rain “middle”	-1.131	0.283	-4	< 0.001
Rain “high”	-2.406	0.906	-2.65	< 0.01

Negative binomial dispersion parameter α = 0.10125 (SE = 0.0047812). ns: not significant

As *Ae. albopictus* has a seasonal activity pattern, it is not surprising that egg densities also varied between months with highest numbers found in August (Fig. 8 and Table 1).

The variable “rain” that was the cumulative precipitation over the week preceding trap replacement had three levels; “low”, “middle” and “high” with 0–104 mm, over 104 to 209 mm and over 209 to 314 mm, respectively. The categories were chosen because visual inspection of the data suggested a non-linear relationship between egg numbers and total rainfall (Fig. 8).

Intriguingly, rain was negatively associated with the presence of eggs in the traps (Table 1 and Fig. 8). This means that egg numbers were higher when the week preceding trap replacement was drier. In contrast to cumulative precipitation, the model did not improve by including temperature as an explanatory variable.

For the sake of comparability with other related studies, egg numbers are also presented as mean number of eggs over a period of four consecutive weeks, split by districts (Fig. 2). For this descriptive analysis, data from all traps were included. In agreement with the analysis above, there is a general trend in increasing relative egg densities over the years with a sharp increase in egg densities in 2012 compared to the preceding years. Mean densities also reflect the spatial gradient from South to North with highest densities in the district of Mendrisio and the lowest ones in the districts of Bellinzona and Riviera.

## Discussion

Since its first detection in 2003 at Ticino’s southern tip to Italy *Ae. albopictus* has continuously spread north across the lower valleys, mainly along the trans-European motorway E35. This trend continues as witnessed both by a growing infested area, with a clear South-North gradient in the introduction, spread and establishment of the mosquito.

*Ae. albopictus* arrival in Canton Ticino was not so surprising, given the rapid spread that had previously occurred in most regions in North and Central Italy [18, 19, 39] and the intense road traffic through the trans-European motorway E35 coming from Italy, the most heavily infested country in Europe [21]. On the E35 alone, an estimated daily average of 66,200 vehicles cross the border between Italy and Ticino with over 1 million lorries in 2014 [40]. *Ae. albopictus* introduction with used tyres is considered one of the main pathways globally [41], yet is unlikely to be relevant for Ticino because, to our knowledge, used tyres are not imported into Switzerland. The main pathway of importation is most probably the motorway as the parking areas were the first to be positive for the mosquito and the observed pattern that started the adjacent residential sites have been colonised from there. Industrial areas nearby, where goods from abroad are imported and where many cross-border commuters work, are also likely to be key ports of passive mosquito introductions. From there the mosquito probably might have spread into neighbouring residential areas where *Ae. albopictus* has presumably managed to establish as, perhaps, many breeding sites are available in residential areas and the heating of buildings might create a more suitable microclimate.

Due to the heavy traffic crossing the Swiss-Italian border, re-infestation is likely to be continuously taking place in addition to the already established local *Ae. albopictus* populations. Once established, it is conceivable that local road traffic fuelled further dispersal of the mosquito, which, for example, could explain the rapid extension of *Ae. albopictus* in 2012. In fact between 2011 and 2012 no significant climatic changes occurred as far as we are aware. Even if this mosquito species seems not to fly long distances as field studies suggest [14], the mosquito will still actively disperse albeit at slower speed. In that sense, higher mosquito densities increase the chance of the mosquito spreading into adjacent areas. In contrast, when a built area is isolated, as it was the case for Locarno airport, it might be more difficult for a mosquito to gain ground in residential areas and it is also more easily intercepted by control measures. In 2008 and 2009 when the mosquito reappeared around the airport it was probably linked to touristic activities.

Given that Ticino shows the climatic conditions for an establishment of *Ae. albopictus* [42], it is not surprising to see a similar trend as in other areas in Europe south of the Alps, mainly along the Mediterranean coast [43, 44], and in comparable areas in the USA [15]. Generally, the annual mean temperature of 12.5 °C recorded in the study area exceeds the suggested 11 °C threshold for *Ae. albopictus* development [45]. Similarly, a mean temperature of 2.9 °C in January, which is above the suggested thresholds of 0 °C [45], would also not prevent eggs from overwintering. Mean precipitation of 1789 mm during the whole year and 857 mm during the mosquito season between May and September also provide sufficient breeding sites [46]. However, a closer look at the local weather conditions suggests an impact on the speed of how *Ae. albopictus* infests new areas. In areas with an annual mean of 12 °C, including Biasca (Riviera district), Magadino/Cadenazzo and Stabio (Locarno and Mendrisio districts, respectively), the spread and following establishment were delayed. Here, the minimum mean January temperatures varied between 1.5 °C and 2.2 °C. At Magadino/Cadenazzo (Locarno district) and Stabio (Mendrisio district) the minimum temperatures were frequently even below -10 °C. Minus 10 °C is considered the absolute minimum temperature for the survival of overwintering eggs [37, 38]. This may explain why the mosquito’s spread is slow or absent in those regions.

In Biasca (Riviera district), the northernmost surveyed area, *Ae. albopictus* was repeatedly detected at the end of the survey season (August- September) in several years, yet there are no signs of firm establishment even though weather conditions are comparable to Magadino/Cadenazzo and Stabio where the mosquito is, indeed, established. A possible explanation could be that there is less importation of adults in vehicles.

The survey focused on areas below 400 m a.s.l. Regions at higher altitudes were included if *Ae. albopictus* was spreading into the neighbouring valley floors [27]. The mosquito was observed there even though it has difficulty to establish, probably because of unfavourable weather conditions for establishment and low road traffic.

Even though estimation of establishment and overwintering capacity using ovitrap data is not optimal because of the competition with existing breeding sites, our detailed analysis showed a clear dynamic trend. *Ae. albopictus* appeared sporadically in places and then became more and more present in the same spot the following years, suggesting gradual establishment of locally reproducing populations that manage to overwinter. However, our observations do not allow an estimate of the proportion of the *Ae. albopictus* population in Ticino that is stable and what proportion is continuously displaced passively. A better understanding of the population dynamics and a better knowledge of the threshold required for the establishment of a population are needed to assess the potential for further establishment and to improve targeted mosquito surveillance. Nevertheless, considering that low temperatures reduce mosquito establishment, whereas intense road traffic and habitat suitability of the residential areas appear to favour the mosquitoes arrival and establishment, our observations help in setting priorities in the survey and control measures. Therefore, we suggest prioritising residential areas as well as areas where traffic and human population densities are higher.

The onset of seasonal activity of *Ae. albopictus* occurred in mid- to end-April with the diapause exit, when day length and mean temperature were 11–11.5 h and 12.3 °C, respectively. This corresponds to what was reported from Rome (11 to 11.5 h daylight with mean temperatures of 10 to 11 °C) [47], where the maximum egg counts were observed in August, when the mean temperature was about 21.1 °C. This corresponds to what was reported in other Italian regions such as Emilia-Romagna [48]. In Ticino, *Ae. albopictus* was active until mid-November when day length corresponded to 10 h of daylight and when the mean temperature was about 8 °C. To date, no winter activity has been observed, in contrast to what was reported from slightly warmer regions such as Emilia-Romagna and Rome with an annual mean temperature of 12.5 °C [49] and 15.7 °C [50], respectively. There, freshly deposited eggs and adults were also reported during winter [32, 48]. The trend of incessant and prolonged diapause with higher latitude is a phenomenon also observed in northern America [51]. Generally, the pattern observed here corresponds to the Italian Province of Trento, another sub-Alpine area where *Ae. albopictus* is overwintering as dormant eggs and where climatic conditions are similar to Ticino [52].

To detect *Ae. albopictus* and estimate its relative population size, ovitraps were the method of choice because these traps are sensitive at low mosquito densities [3], relatively cheap and require little maintenance. Some authors have, however, raised concerns over the validity of ovitraps for relative density estimates due to their competing nature with existing breeding sites (e.g. [53]) and because a single female may deposit its eggs in multiple sites [54], a behaviour known as skip oviposition. The use of indices and overall mean values of non-normally distributed egg counts rather than working directly with the actual data as done in the present study may explain the different conclusions among studies. Intriguingly, in support of our study, Carrieri et al. [55, 56] found that egg counts, estimated by means of ovitrap monitoring, were a reliable proxy for the mean number of biting females per unit area as well as larval productivity. In an attempt to learn more about the dynamics of relative mosquito population densities we therefore decided to include in our analysis also actual egg counts rather than reducing the whole data set to a mere presence-absence table. Considering the highly over dispersed data set owing to high numbers of empty traps and to account for correlation due to repeated measures in the same trap over time we have chosen to model the data with a GLMM with a negative binomial link function. Initially we had also looked at a model with a zero inflated negative binomial distribution, but we did not see an improvement in fitting the data.

We also found that more eggs were laid when weekly accumulated rainfall was lower. On the one hand, this may be explained by breeding sites becoming less frequent during drier periods making the ovitraps more attractive to egg laying females, so that egg counts are an overestimate of relative abundance. On the other hand, intense precipitation reduces abundance over short periods of host seeking females [57]. In line with the latter, it may be argued that the artificial containers in which *Ae. albopictus* is mainly breeding are still present during dry periods because under these circumstances residents still water flowers or store water in tanks in their gardens.

The observations made in the current study together with the overall trend in Europe [21] as well as models predicting habitat suitability under present and future climate scenarios [42, 58] suggest that *Ae. albopictus* will continue to spread from the Mediterranean regions further into northern Europe by passive transport. Indeed, *Ae. albopictus* is already spotted more frequently along the E35 north of the Alps both in Switzerland [59, 60] and Germany [23–25]. *Ae. albopictus* is expected to infest areas that are already climatically suitable such as the region around the Lake Geneva [42], given the area is also well connected through major traffic routes to the South of France, where the mosquito is well established. In addition, although models consider environmental mean temperatures [42, 44, 46, 61], urban settlements offer microclimates that are warmer in winters than the ones recorded by weather stations because of heating. Urban areas could favour the *Ae. albopictus* overwintering and allow its establishment in regions not considered suitable so far.

In Europe, autochthonous cases of dengue and chikungunya appeared shortly after the mosquito’s peak season (late summer to mid autumn) when mosquito densities were high enough and temperatures still favourable for viral replication in the mosquito [6–13]. While egg count is a good indicator of the presence of *Ae. albopictus*, and to some extent also of its relative density, it would be useful if such data could also be used in more detail to make inference of the risk for disease transmission and outbreaks. Carrieri et al. [56] estimated epidemiological thresholds, modelling vectorial capacity calibrated by egg numbers against the number of host seeking females using landing catches. Epidemic thresholds should still be estimated with parameters measured in the Canton of Ticino following e.g. the Italian model by including other factors such as the number of infected people returning from endemic areas [62–64].

## Conclusions

*Ae. albopictus* has firmly established in Ticino and is continuously expanding its range from South to North. Though the local patterns may differ due to variations in traffic load and local climatic conditions, our results suggest a more universal trend in that *Ae. albopictus* continues spreading and increasing in densities, which is a call for continued surveillance.

## Additional files

Additional file 1: Annual trapping scheme for the surveys between 2003 and 2014. The numbers indicate the collection round in each year. The collection rounds included in the analysis of the establishment and overwintering of *Ae. albopictus* are in bold. The numbers in brackets indicate collection rounds in which traps were set and replaced but the slats were not inspected. (DOC 28 kb) Additional file 2: Meteorological stations in the study areas and weather conditions between 2006 and 2014. *AnnT* ^*m*^: Annual mean temperature; *JanT* ^*m*^: mean temperature of the coldest month; *SumT* ^*m*^; mean temperature of the survey season (May-September); DT^min<-10^°^C^: number of days with an average temperature below -10 °C. Agrometeo data were retrieved from www.agrometeo.ch and MeteoSwiss data from www.meteoswiss.ch. (DOCX 15 kb) Additional file 3:
*Aedes albopictus* presence in Canton Ticino (southern Switzerland) over the years. The file contains a series of maps for each year from 2003 to 2014. Each map shows for a particular year where ovitraps were positive or negative for *Ae. albopictus* eggs. A dot represents an ovitrap and is colour-coded according to its status; green indicates the trap was always negative, red shows that eggs were found at least once, purple indicates seasonal establishment (i.e. the trap was repeatedly positive over at least 3 months), and blue indicates the overwintering (i.e. the trap was positive the last control round of a year and the first control round of the following one). Map layers were purchased from the Swiss Federal Office of Topography. (ZIP 20813 kb) Additional file 4: Result summary of the Ticino surveillance programme from 2003 to 2014. Only data from available slats are reported. The area covered by the surveillance programme was estimated by adding the surface area of a virtual 250 m by 250 m grid that covered the ovitraps. (DOC 25 kb) Additional file 5: Geographical distribution of ovitraps in the Canton of Ticino between 2003 and 2014. The red dots represent the 46 sentinel ovitraps included in the analysis of the egg counts between 2006 and 2014. The thick and thin red lines indicate the national and district borders, respectively. The yellow line shows the motorways, including the trans-European motorway E35 running from South to North. Light blue shaded areas are lakes. CH: Switzerland; IT: Italy. (JPG 1055 kb)
