# Supplementary material for: Spread and establishment of Aedes albopictus in southern Switzerland between 2003 and 2014: an analysis of oviposition data and weather conditions
Source: Parasit Vectors. 2016 May 26;9:304. doi: 10.1186/s13071-016-1577-3 (PMC4882898; doi:10.1186/s13071-016-1577-3)
Supplement: Additional file 1: — Annual trapping scheme for the surveys between 2003 and 2014. The numbers indicate the collection round in each year. The collection rounds included in the analysis of the establishment and overwintering of Ae. albopictus are in bold. The numbers in brackets indicate collection rounds in which traps were set and replaced but the slats were not inspected. (DOC 28 kb) [file 13071_2016_1577_MOESM1_ESM.doc]

**Additional file 1 – Annual trapping scheme for the surveys between 2003 and 2014**

The numbers indicate the collection round in each year. The collection rounds included in the analysis of the establishment and overwintering of *Ae. albopictus* are in bold. The numbers in brackets indicate collection rounds in which traps were set and replaced but the slats were not inspected.
