# Supplementary material for: Spread and establishment of Aedes albopictus in southern Switzerland between 2003 and 2014: an analysis of oviposition data and weather conditions
Source: Parasit Vectors. 2016 May 26;9:304. doi: 10.1186/s13071-016-1577-3 (PMC4882898; doi:10.1186/s13071-016-1577-3)
Supplement: Additional file 2: — Meteorological stations in the study areas and weather conditions between 2006 and 2014. AnnT m: Annual mean temperature; JanT m: mean temperature of the coldest month; SumT m; mean temperature of the survey season (May-September); DTmin<-10°C: number of days with an average temperature below -10 °C. Agrometeo data were retrieved from www.agrometeo.ch and MeteoSwiss data from www.meteoswiss.ch. (DOCX 15 kb) [file 13071_2016_1577_MOESM2_ESM.docx]

**Additional file 2 – Meteorological stations in the study areas and weather conditions between 2006 and 2014**

*AnnT^m^*: Annual mean temperature; *JanT^m^*: mean temperature of the coldest month; *SumT^m^*; mean temperature of the survey season (May-September); DT^min<-10°C^: number of days with an average temperature below -10 °C. Agrometeo data were retrieved from [www.agrometeo.ch](www.agrometeo.ch%20) and MeteoSwiss data from [www.meteoswiss.ch](http://www.meteoswiss.ch).
