# Supplementary figures and images for: Spread and establishment of Aedes albopictus in southern Switzerland between 2003 and 2014: an analysis of oviposition data and weather conditions
Source: Parasit Vectors. 2016 May 26;9:304. doi: 10.1186/s13071-016-1577-3 (PMC4882898; doi:10.1186/s13071-016-1577-3)

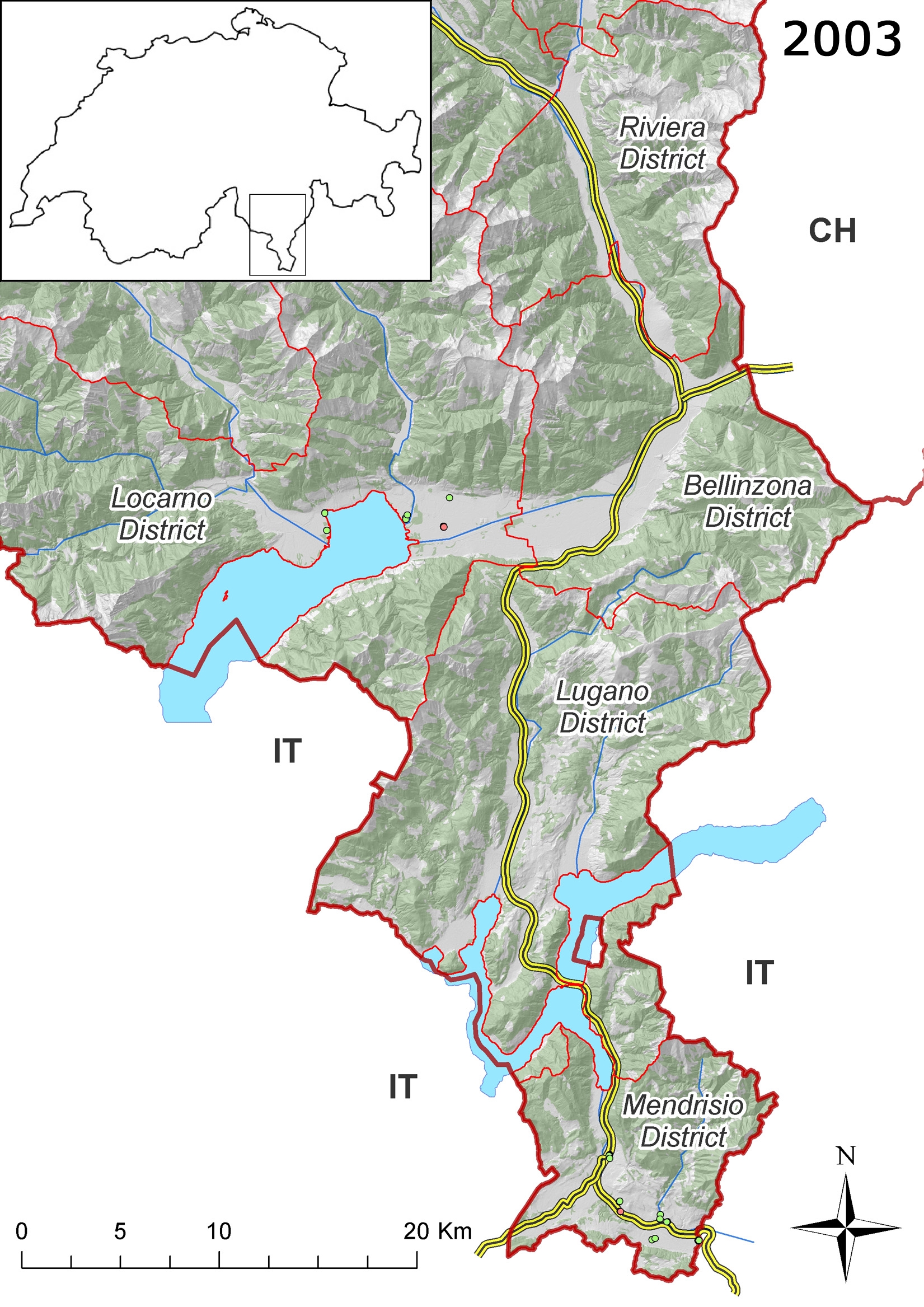

Supplement: Additional file 3: — Aedes albopictus presence in Canton Ticino (southern Switzerland) over the years. The file contains a series of maps for each year from 2003 to 2014. Each map shows for a particular year where ovitraps were positive or negative for Ae. albopictus eggs. A dot represents an ovitrap and is colour-coded according to its status; green indicates the trap was always negative, red shows that eggs were found at least once, purple indicates seasonal establishment (i.e. the trap was repeatedly positive over at least 3 months), and blue indicates the overwintering (i.e. the trap was positive the last control round of a year and the first control round of the following one). Map layers were purchased from the Swiss Federal Office of Topography. (ZIP 20813 kb) [file 13071_2016_1577_MOESM3_ESM.zip › additional file 3/Flacio et al. Spread and establishment of Ae. albopictus_Additional file 3_2003.JPG]

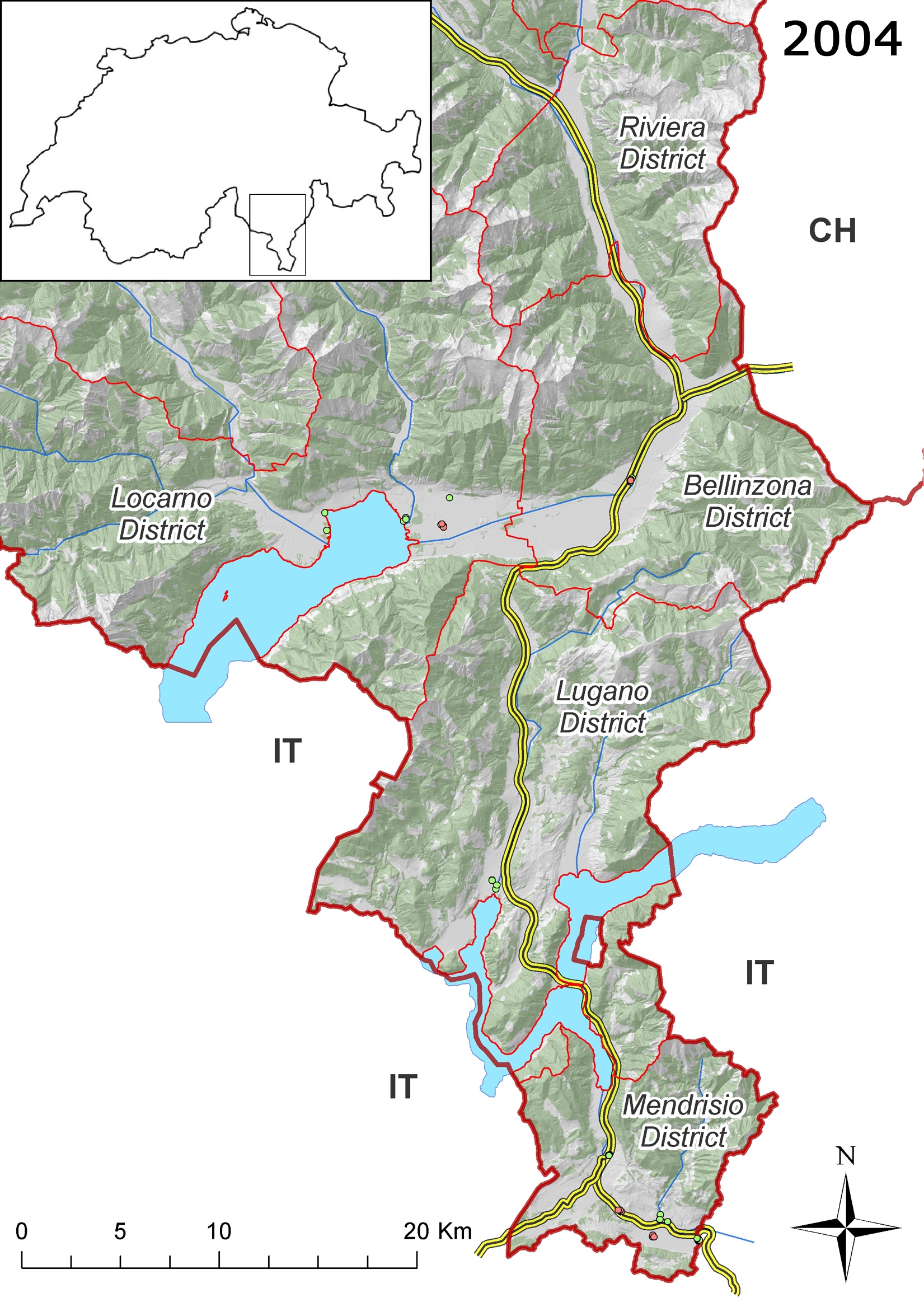

Supplement: Additional file 3: — Aedes albopictus presence in Canton Ticino (southern Switzerland) over the years. The file contains a series of maps for each year from 2003 to 2014. Each map shows for a particular year where ovitraps were positive or negative for Ae. albopictus eggs. A dot represents an ovitrap and is colour-coded according to its status; green indicates the trap was always negative, red shows that eggs were found at least once, purple indicates seasonal establishment (i.e. the trap was repeatedly positive over at least 3 months), and blue indicates the overwintering (i.e. the trap was positive the last control round of a year and the first control round of the following one). Map layers were purchased from the Swiss Federal Office of Topography. (ZIP 20813 kb) [file 13071_2016_1577_MOESM3_ESM.zip › additional file 3/Flacio et al. Spread and establishment of Ae. albopictus_Additional file 3_2004.JPG]

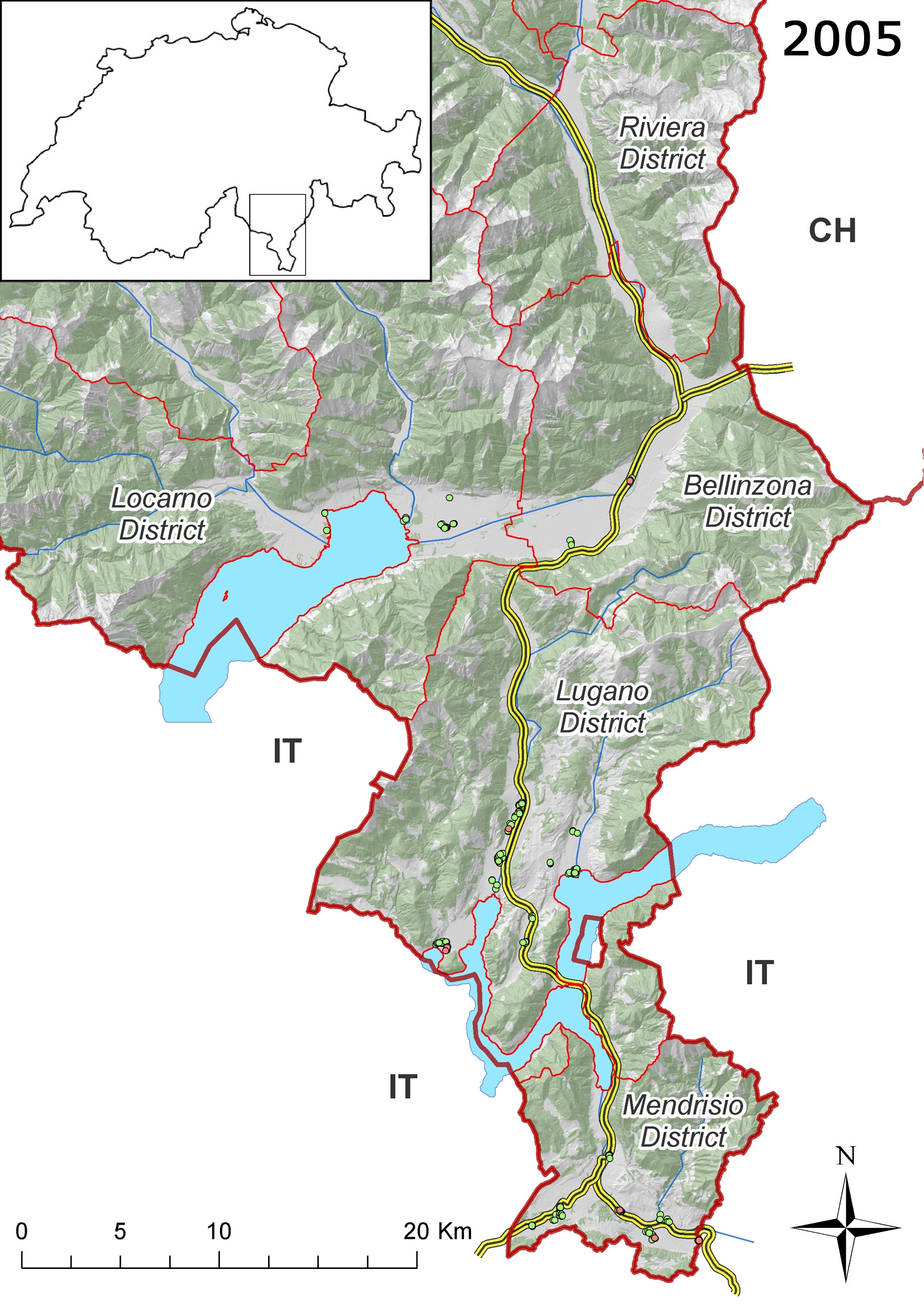

Supplement: Additional file 3: — Aedes albopictus presence in Canton Ticino (southern Switzerland) over the years. The file contains a series of maps for each year from 2003 to 2014. Each map shows for a particular year where ovitraps were positive or negative for Ae. albopictus eggs. A dot represents an ovitrap and is colour-coded according to its status; green indicates the trap was always negative, red shows that eggs were found at least once, purple indicates seasonal establishment (i.e. the trap was repeatedly positive over at least 3 months), and blue indicates the overwintering (i.e. the trap was positive the last control round of a year and the first control round of the following one). Map layers were purchased from the Swiss Federal Office of Topography. (ZIP 20813 kb) [file 13071_2016_1577_MOESM3_ESM.zip › additional file 3/Flacio et al. Spread and establishment of Ae. albopictus_Additional file 3_2005.JPG]

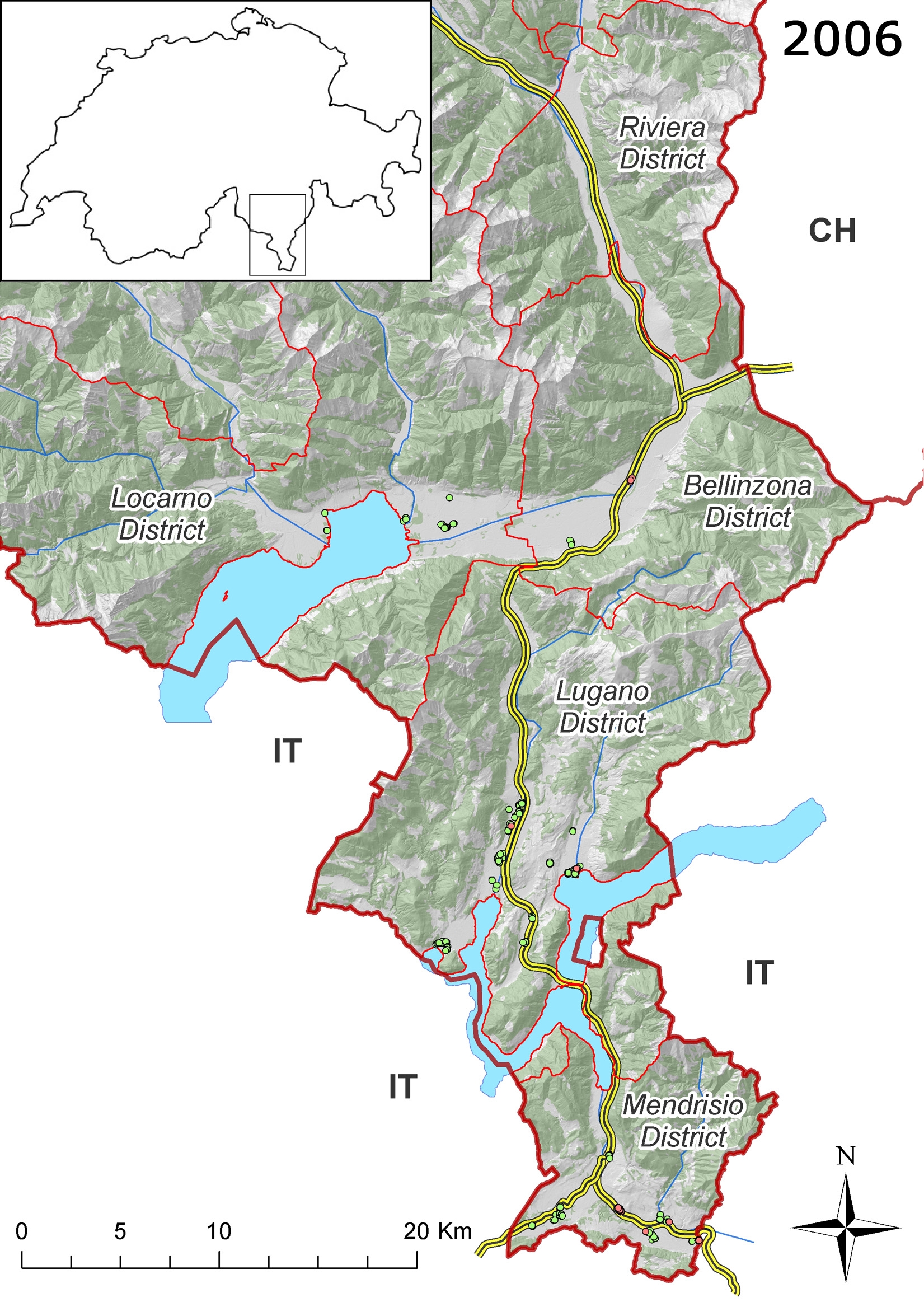

Supplement: Additional file 3: — Aedes albopictus presence in Canton Ticino (southern Switzerland) over the years. The file contains a series of maps for each year from 2003 to 2014. Each map shows for a particular year where ovitraps were positive or negative for Ae. albopictus eggs. A dot represents an ovitrap and is colour-coded according to its status; green indicates the trap was always negative, red shows that eggs were found at least once, purple indicates seasonal establishment (i.e. the trap was repeatedly positive over at least 3 months), and blue indicates the overwintering (i.e. the trap was positive the last control round of a year and the first control round of the following one). Map layers were purchased from the Swiss Federal Office of Topography. (ZIP 20813 kb) [file 13071_2016_1577_MOESM3_ESM.zip › additional file 3/Flacio et al. Spread and establishment of Ae. albopictus_Additional file 3_2006.JPG]

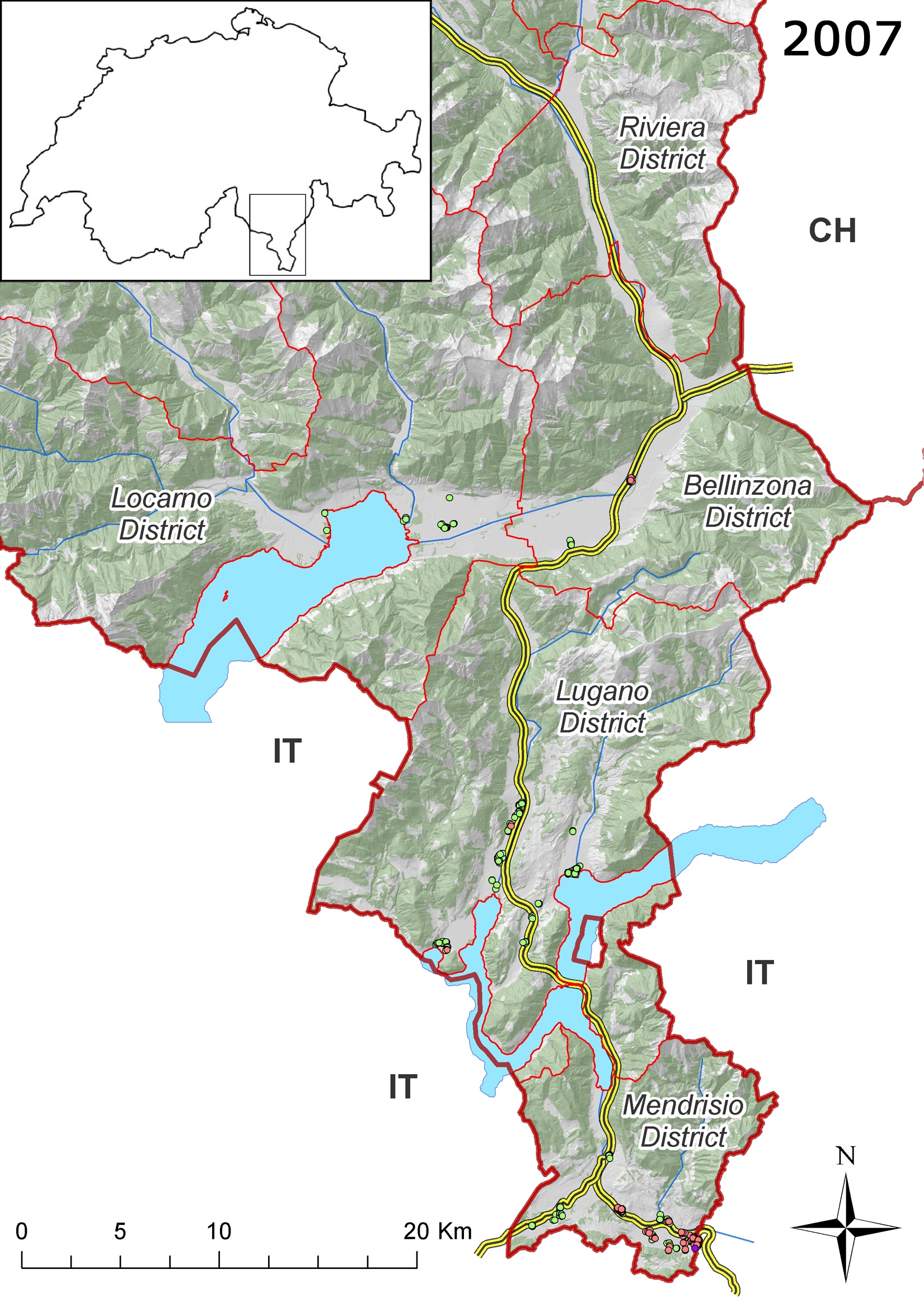

Supplement: Additional file 3: — Aedes albopictus presence in Canton Ticino (southern Switzerland) over the years. The file contains a series of maps for each year from 2003 to 2014. Each map shows for a particular year where ovitraps were positive or negative for Ae. albopictus eggs. A dot represents an ovitrap and is colour-coded according to its status; green indicates the trap was always negative, red shows that eggs were found at least once, purple indicates seasonal establishment (i.e. the trap was repeatedly positive over at least 3 months), and blue indicates the overwintering (i.e. the trap was positive the last control round of a year and the first control round of the following one). Map layers were purchased from the Swiss Federal Office of Topography. (ZIP 20813 kb) [file 13071_2016_1577_MOESM3_ESM.zip › additional file 3/Flacio et al. Spread and establishment of Ae. albopictus_Additional file 3_2007.JPG]

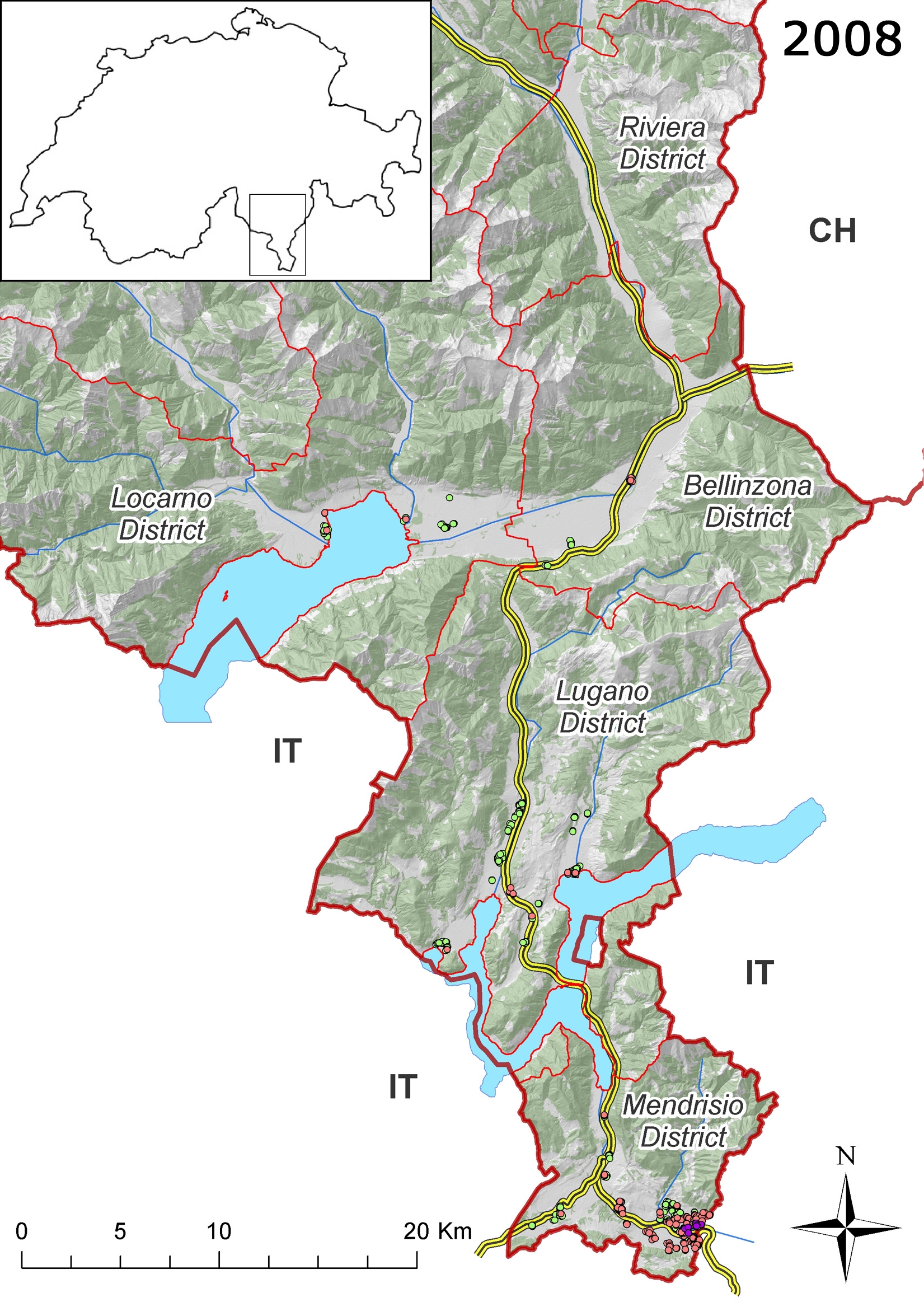

Supplement: Additional file 3: — Aedes albopictus presence in Canton Ticino (southern Switzerland) over the years. The file contains a series of maps for each year from 2003 to 2014. Each map shows for a particular year where ovitraps were positive or negative for Ae. albopictus eggs. A dot represents an ovitrap and is colour-coded according to its status; green indicates the trap was always negative, red shows that eggs were found at least once, purple indicates seasonal establishment (i.e. the trap was repeatedly positive over at least 3 months), and blue indicates the overwintering (i.e. the trap was positive the last control round of a year and the first control round of the following one). Map layers were purchased from the Swiss Federal Office of Topography. (ZIP 20813 kb) [file 13071_2016_1577_MOESM3_ESM.zip › additional file 3/Flacio et al. Spread and establishment of Ae. albopictus_Additional file 3_2008.JPG]

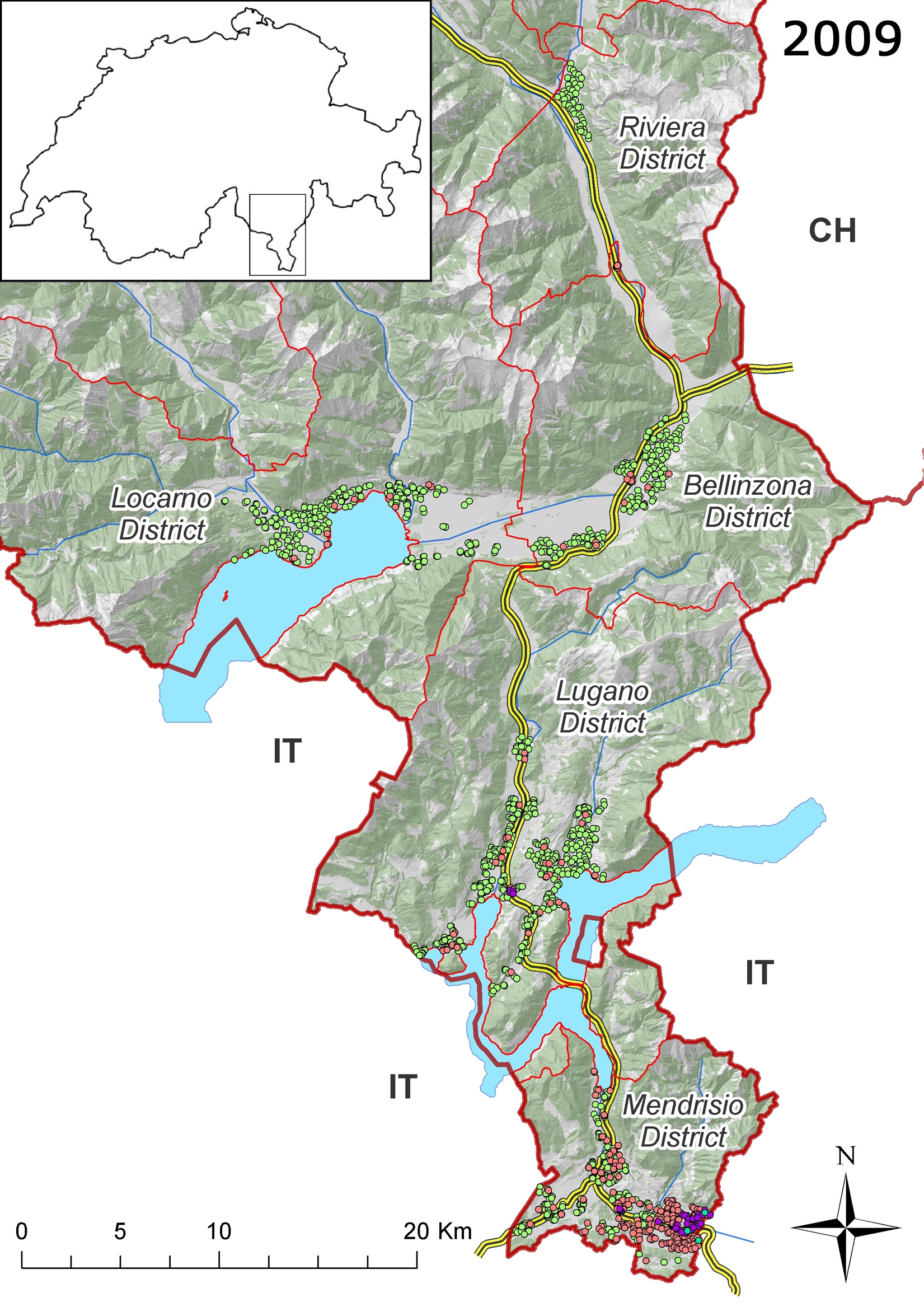

Supplement: Additional file 3: — Aedes albopictus presence in Canton Ticino (southern Switzerland) over the years. The file contains a series of maps for each year from 2003 to 2014. Each map shows for a particular year where ovitraps were positive or negative for Ae. albopictus eggs. A dot represents an ovitrap and is colour-coded according to its status; green indicates the trap was always negative, red shows that eggs were found at least once, purple indicates seasonal establishment (i.e. the trap was repeatedly positive over at least 3 months), and blue indicates the overwintering (i.e. the trap was positive the last control round of a year and the first control round of the following one). Map layers were purchased from the Swiss Federal Office of Topography. (ZIP 20813 kb) [file 13071_2016_1577_MOESM3_ESM.zip › additional file 3/Flacio et al. Spread and establishment of Ae. albopictus_Additional file 3_2009.JPG]

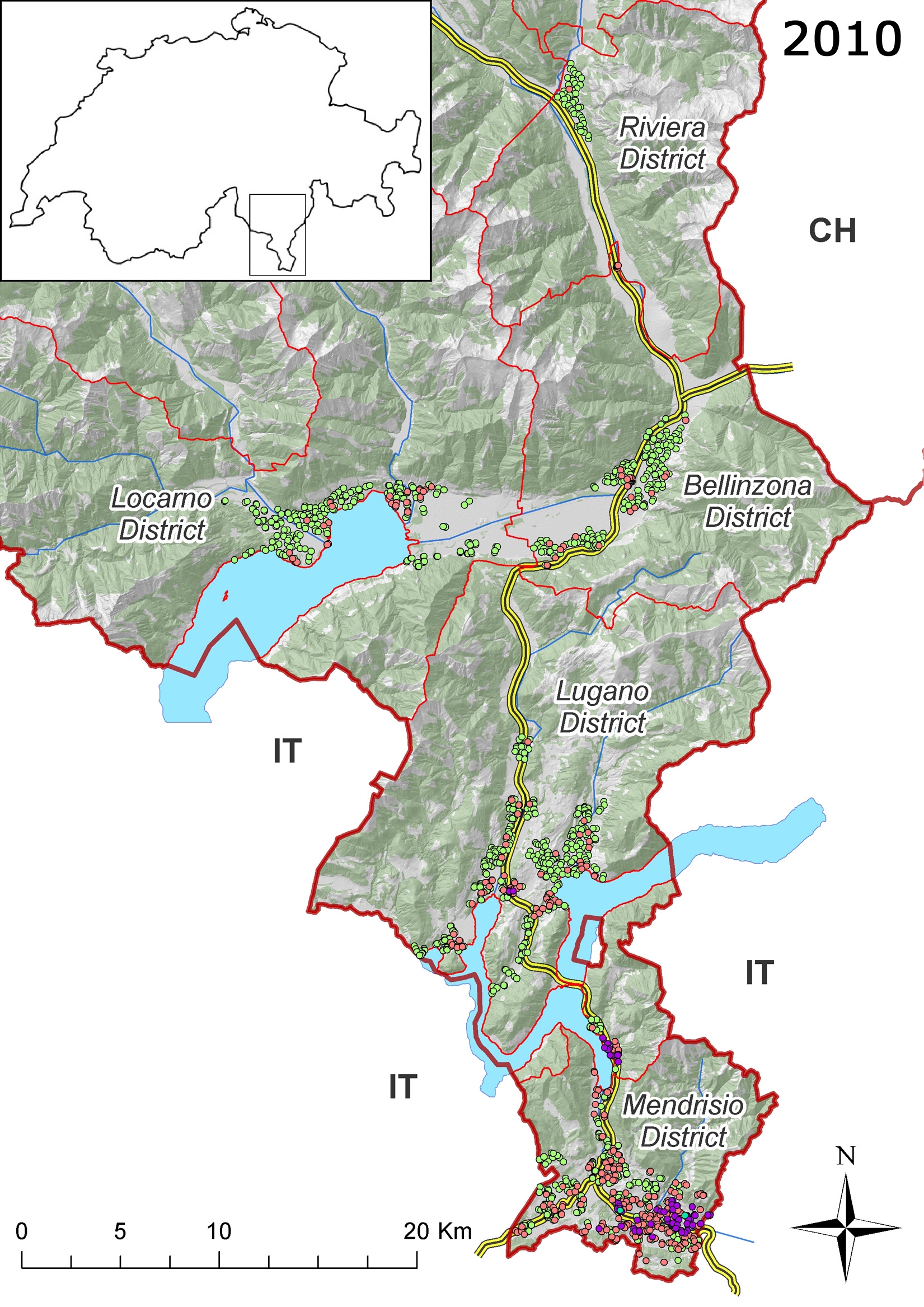

Supplement: Additional file 3: — Aedes albopictus presence in Canton Ticino (southern Switzerland) over the years. The file contains a series of maps for each year from 2003 to 2014. Each map shows for a particular year where ovitraps were positive or negative for Ae. albopictus eggs. A dot represents an ovitrap and is colour-coded according to its status; green indicates the trap was always negative, red shows that eggs were found at least once, purple indicates seasonal establishment (i.e. the trap was repeatedly positive over at least 3 months), and blue indicates the overwintering (i.e. the trap was positive the last control round of a year and the first control round of the following one). Map layers were purchased from the Swiss Federal Office of Topography. (ZIP 20813 kb) [file 13071_2016_1577_MOESM3_ESM.zip › additional file 3/Flacio et al. Spread and establishment of Ae. albopictus_Additional file 3_2010.JPG]

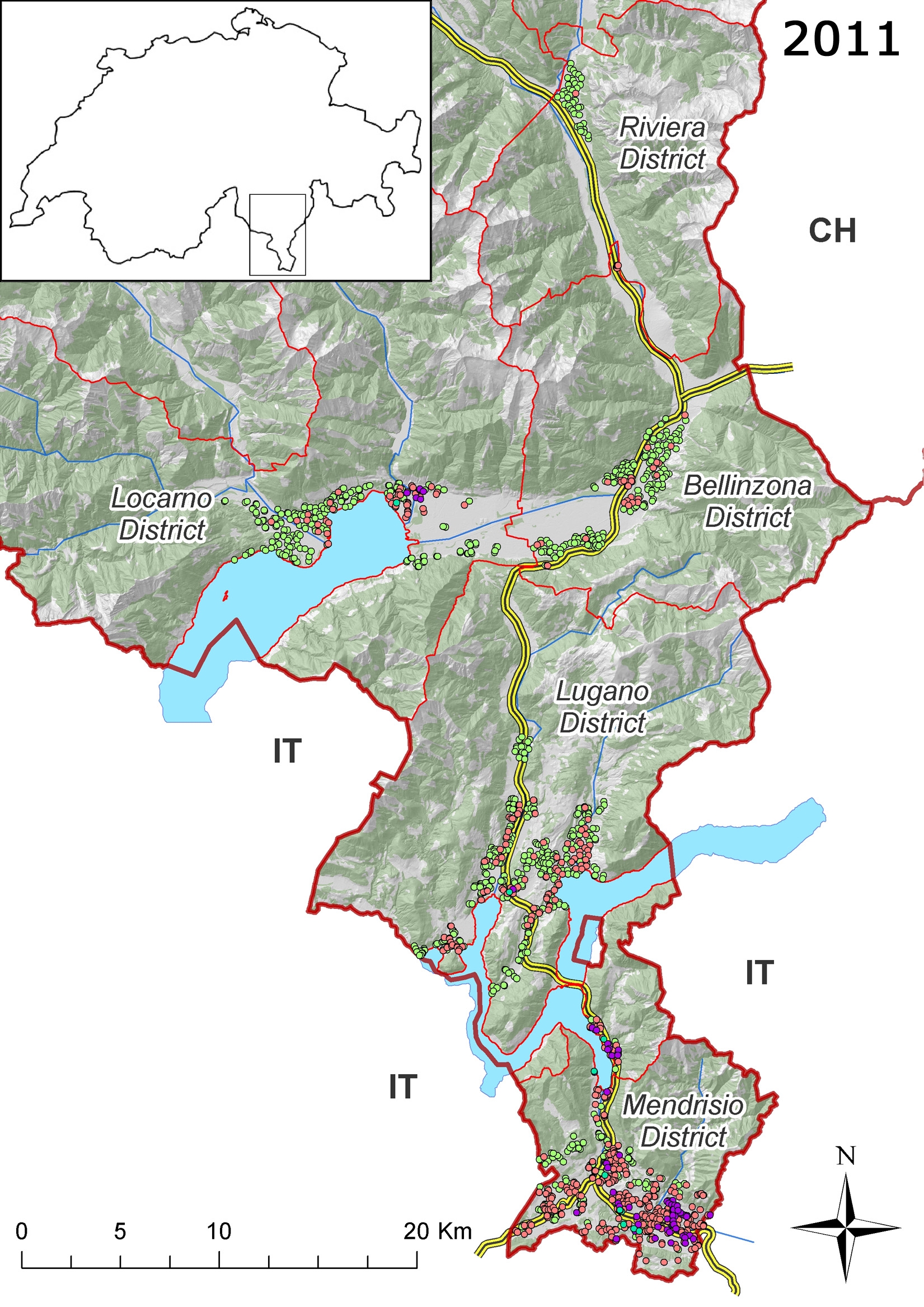

Supplement: Additional file 3: — Aedes albopictus presence in Canton Ticino (southern Switzerland) over the years. The file contains a series of maps for each year from 2003 to 2014. Each map shows for a particular year where ovitraps were positive or negative for Ae. albopictus eggs. A dot represents an ovitrap and is colour-coded according to its status; green indicates the trap was always negative, red shows that eggs were found at least once, purple indicates seasonal establishment (i.e. the trap was repeatedly positive over at least 3 months), and blue indicates the overwintering (i.e. the trap was positive the last control round of a year and the first control round of the following one). Map layers were purchased from the Swiss Federal Office of Topography. (ZIP 20813 kb) [file 13071_2016_1577_MOESM3_ESM.zip › additional file 3/Flacio et al. Spread and establishment of Ae. albopictus_Additional file 3_2011.JPG]

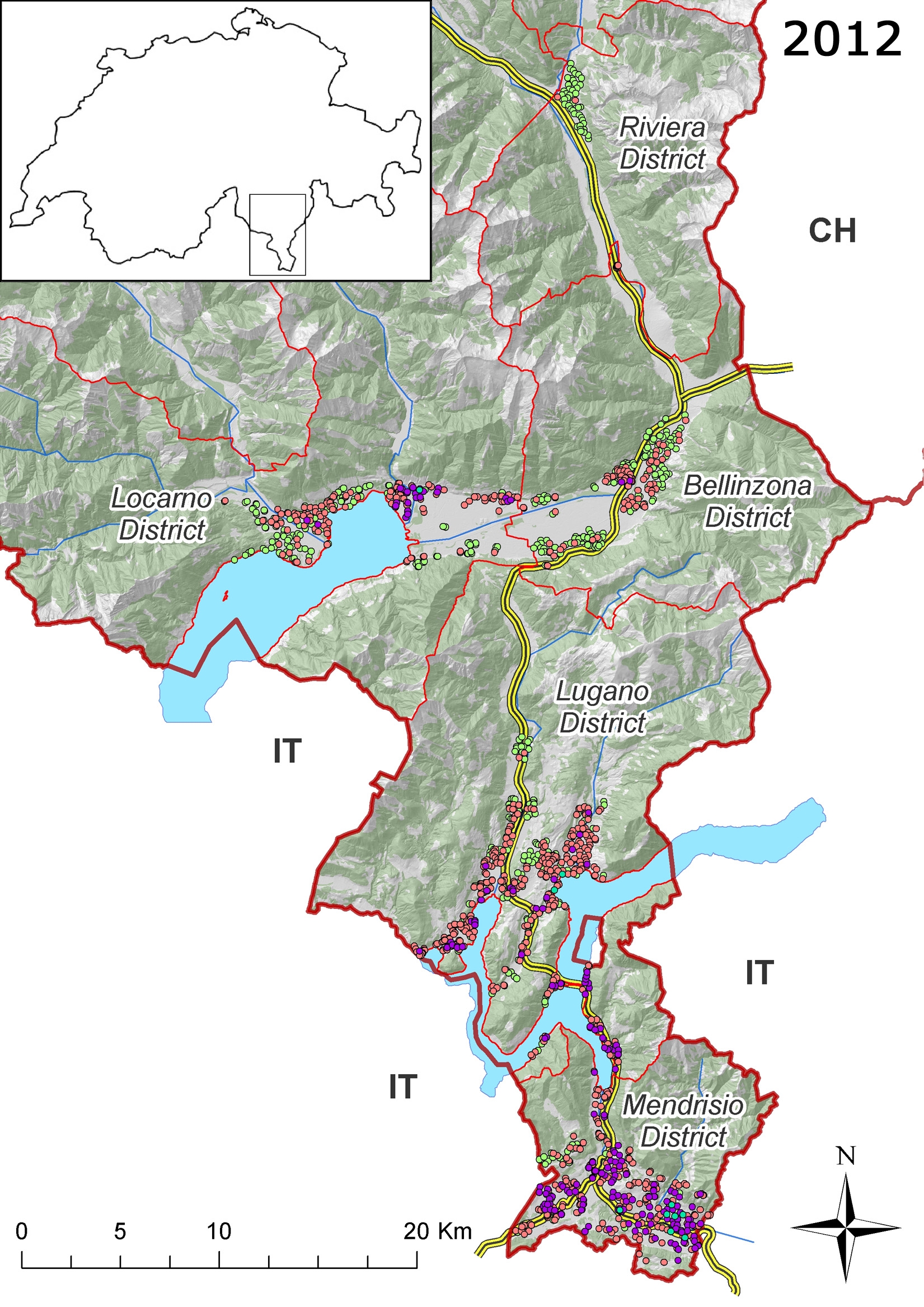

Supplement: Additional file 3: — Aedes albopictus presence in Canton Ticino (southern Switzerland) over the years. The file contains a series of maps for each year from 2003 to 2014. Each map shows for a particular year where ovitraps were positive or negative for Ae. albopictus eggs. A dot represents an ovitrap and is colour-coded according to its status; green indicates the trap was always negative, red shows that eggs were found at least once, purple indicates seasonal establishment (i.e. the trap was repeatedly positive over at least 3 months), and blue indicates the overwintering (i.e. the trap was positive the last control round of a year and the first control round of the following one). Map layers were purchased from the Swiss Federal Office of Topography. (ZIP 20813 kb) [file 13071_2016_1577_MOESM3_ESM.zip › additional file 3/Flacio et al. Spread and establishment of Ae. albopictus_Additional file 3_2012.JPG]

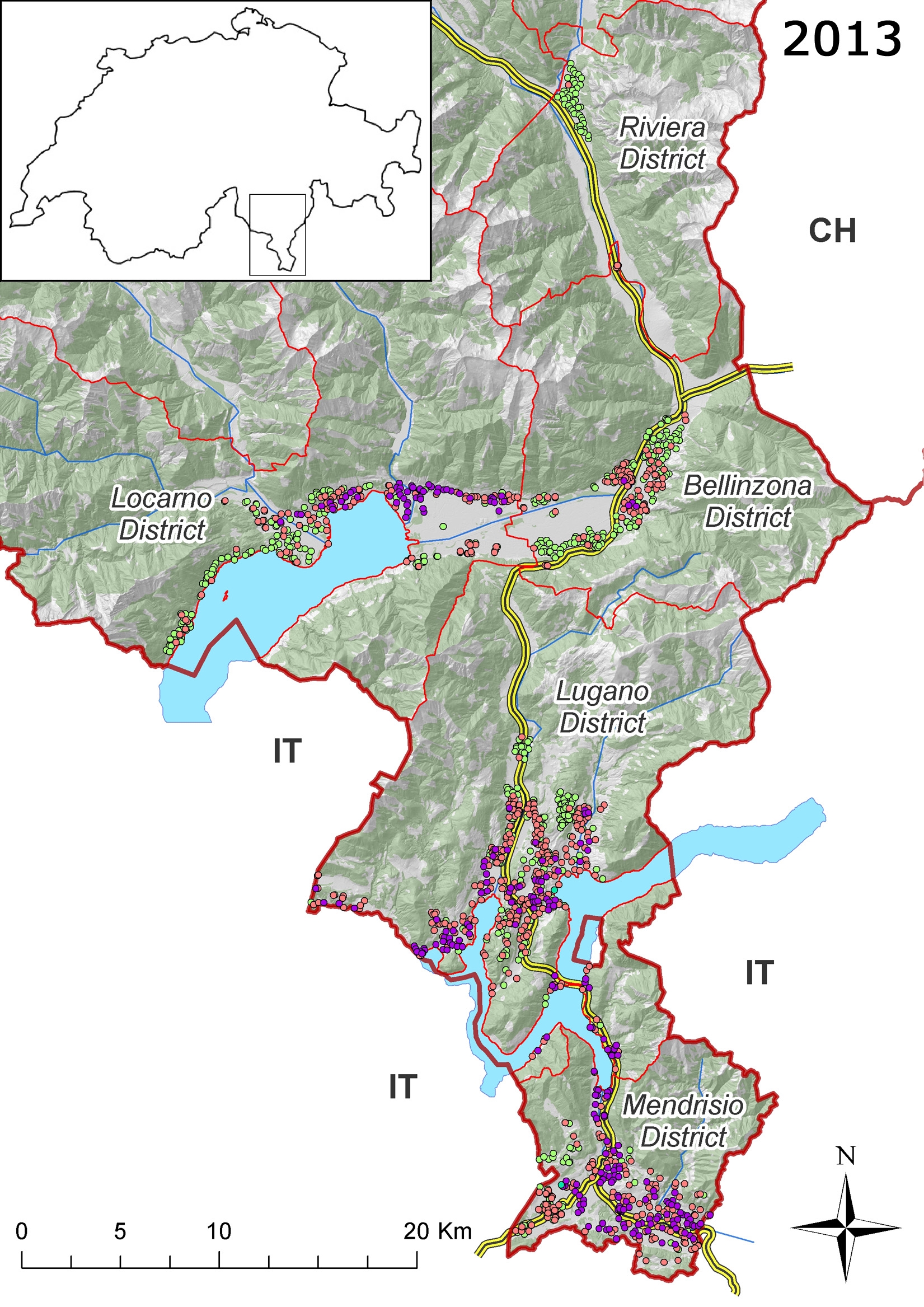

Supplement: Additional file 3: — Aedes albopictus presence in Canton Ticino (southern Switzerland) over the years. The file contains a series of maps for each year from 2003 to 2014. Each map shows for a particular year where ovitraps were positive or negative for Ae. albopictus eggs. A dot represents an ovitrap and is colour-coded according to its status; green indicates the trap was always negative, red shows that eggs were found at least once, purple indicates seasonal establishment (i.e. the trap was repeatedly positive over at least 3 months), and blue indicates the overwintering (i.e. the trap was positive the last control round of a year and the first control round of the following one). Map layers were purchased from the Swiss Federal Office of Topography. (ZIP 20813 kb) [file 13071_2016_1577_MOESM3_ESM.zip › additional file 3/Flacio et al. Spread and establishment of Ae. albopictus_Additional file 3_2013.JPG]

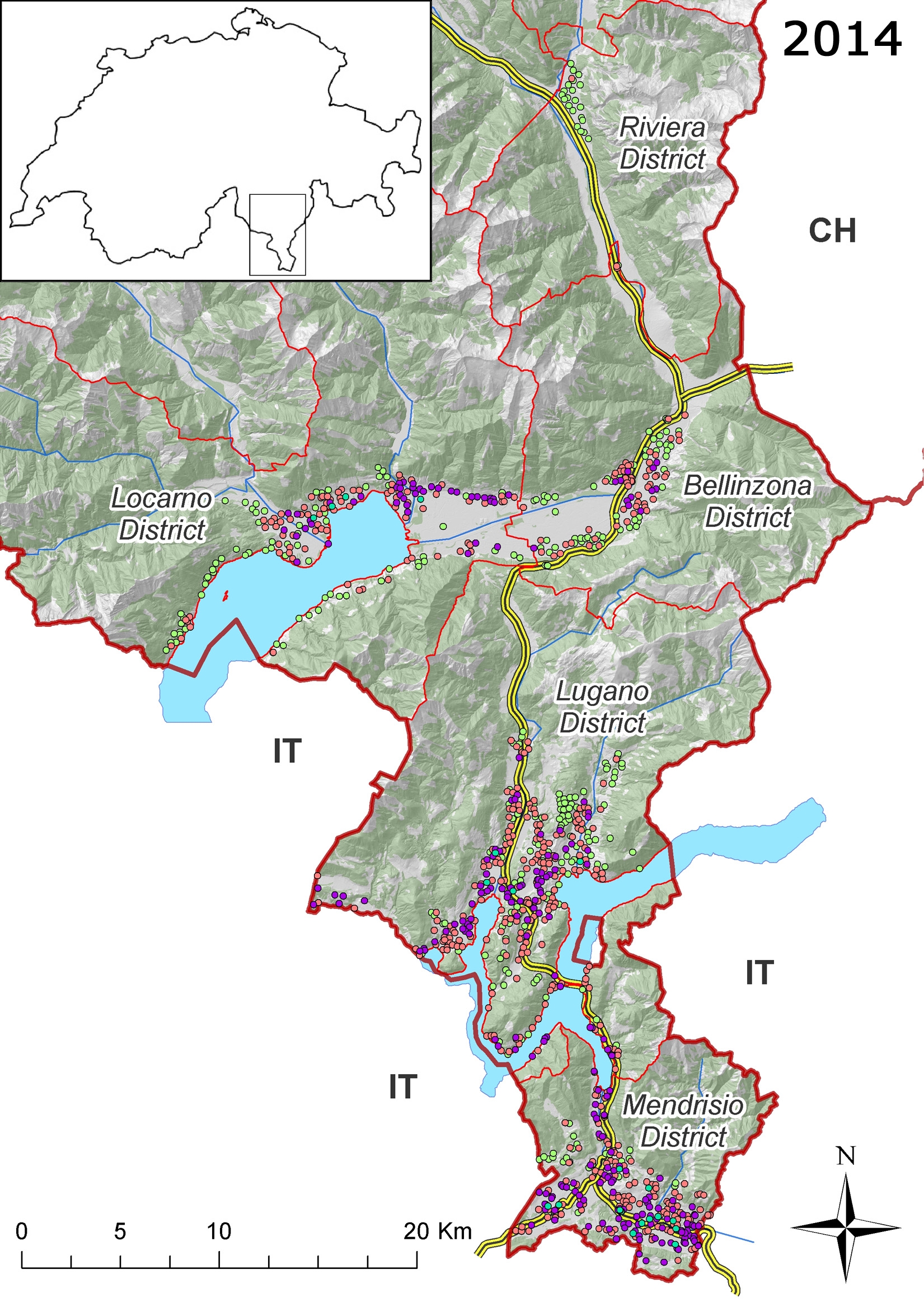

Supplement: Additional file 3: — Aedes albopictus presence in Canton Ticino (southern Switzerland) over the years. The file contains a series of maps for each year from 2003 to 2014. Each map shows for a particular year where ovitraps were positive or negative for Ae. albopictus eggs. A dot represents an ovitrap and is colour-coded according to its status; green indicates the trap was always negative, red shows that eggs were found at least once, purple indicates seasonal establishment (i.e. the trap was repeatedly positive over at least 3 months), and blue indicates the overwintering (i.e. the trap was positive the last control round of a year and the first control round of the following one). Map layers were purchased from the Swiss Federal Office of Topography. (ZIP 20813 kb) [file 13071_2016_1577_MOESM3_ESM.zip › additional file 3/Flacio et al. Spread and establishment of Ae. albopictus_Additional file 3_2014.JPG]

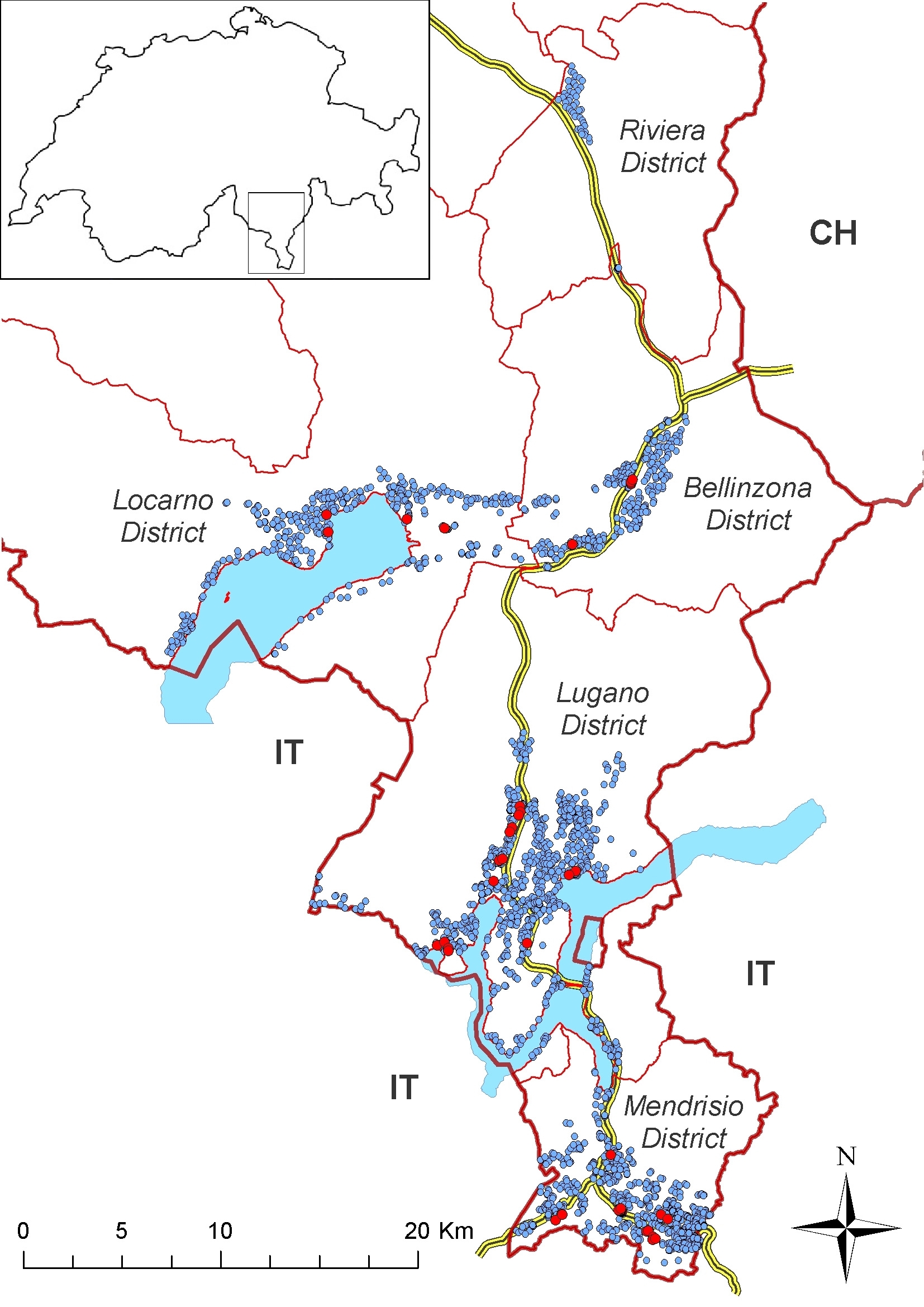

Supplement: Additional file 5: — Geographical distribution of ovitraps in the Canton of Ticino between 2003 and 2014. The red dots represent the 46 sentinel ovitraps included in the analysis of the egg counts between 2006 and 2014. The thick and thin red lines indicate the national and district borders, respectively. The yellow line shows the motorways, including the trans-European motorway E35 running from South to North. Light blue shaded areas are lakes. CH: Switzerland; IT: Italy. (JPG 1055 kb) [file 13071_2016_1577_MOESM5_ESM.jpg]
