# Supplementary material for: Spread and establishment of Aedes albopictus in southern Switzerland between 2003 and 2014: an analysis of oviposition data and weather conditions
Source: Parasit Vectors. 2016 May 26;9:304. doi: 10.1186/s13071-016-1577-3 (PMC4882898; doi:10.1186/s13071-016-1577-3)
Supplement: Additional file 4: — Result summary of the Ticino surveillance programme from 2003 to 2014. Only data from available slats are reported. The area covered by the surveillance programme was estimated by adding the surface area of a virtual 250 m by 250 m grid that covered the ovitraps. (DOC 25 kb) [file 13071_2016_1577_MOESM4_ESM.doc]

**Additional file 4 – Result summary of the Ticino surveillance programme from 2003 to 2014**

Only data from available slats are reported. The area covered by the surveillance programme was estimated by adding the surface area of a virtual 250 m by 250 m grid that covered the ovitraps.
